# Gender Bias in the News: A Scalable Topic Modelling and Visualization Framework

**DOI:** 10.3389/frai.2021.664737

**Published:** 2021-06-16

**Authors:** Prashanth Rao, Maite Taboada

**Affiliations:** Discourse Processing Lab, Department of Linguistics, Simon Fraser University, Burnaby, BC, Canada

**Keywords:** gender bias, news media, machine learning, topic modelling, natural language processing, corpus linguistics

## Abstract

We present a topic modelling and data visualization methodology to examine gender-based disparities in news articles by topic. Existing research in topic modelling is largely focused on the text mining of *closed* corpora, i.e., those that include a fixed collection of composite texts. We showcase a methodology to discover topics via Latent Dirichlet Allocation, which can reliably produce human-interpretable topics over an *open* news corpus that continually grows with time. Our system generates topics, or distributions of keywords, for news articles on a monthly basis, to consistently detect key events and trends aligned with events in the real world. Findings from 2 years worth of news articles in mainstream English-language Canadian media indicate that certain topics feature either women or men more prominently and exhibit different types of language. Perhaps unsurprisingly, topics such as lifestyle, entertainment, and healthcare tend to be prominent in articles that quote more women than men. Topics such as sports, politics, and business are characteristic of articles that quote more men than women. The data shows a self-reinforcing gendered division of duties and representation in society. Quoting female sources more frequently in a caregiving role and quoting male sources more frequently in political and business roles enshrines women’s status as caregivers and men’s status as leaders and breadwinners. Our results can help journalists and policy makers better understand the unequal gender representation of those quoted in the news and facilitate news organizations’ efforts to achieve gender parity in their sources. The proposed methodology is robust, reproducible, and scalable to very large corpora, and can be used for similar studies involving unsupervised topic modelling and language analyses.

## Introduction

Gender equality is one of the UN’s 17 Sustainable Development Goals (United Nations, 2020). By all measures, most societies are far from such equality. Although progress has been made, women are not equally represented in positions of power (OECD, 2020a, OECD, 2020b; Tremblay, 2018; UN Women, 2010); are not equal in science, including in publication metrics (Berenbaum, 2019; King et al., 2017); and do not appear in the news as often as men (Desmond and Danilewicz, 2010; Hong et al., 2020; Macharia, 2015; Shor et al., 2015; Trimble et al., 2021; Van der Pas and Aaldering, 2020). Worryingly, COVID-19 seems to have resulted in set-backs not only in areas such as participation in the workforce and unequal share of unpaid work, but also in media representation (Kassova, 2020a), set-backs which may exacerbate existing stalling trends (England et al., 2020).

The underrepresentation of women in certain areas of the news such as politics, business, or sports is well documented (Power et al., 2019; Kemble, 2020; Thomas et al., 2020; Van der Pas and Aaldering, 2020). There is little large-scale data, however, about representation across entire news organizations, and even less so over a period of time.

In this paper, we tackle one measure of gender equality in the media: the number of times men and women are quoted in news articles. More specifically, we present a scalable topic modelling methodology to explore how many times men and women are quoted across news topics. Our unsupervised large-scale analyses are enhanced with corpus-based studies of the language used in male-prominent and female-prominent articles, thus revealing not only which topics are different, but exactly how they are different in their language.

Our analyses rely on statistics provided by the Gender Gap Tracker,^1^ a purpose-built tool that measures the proportion of male and female sources quoted in mainstream Canadian media in English. The Gender Gap Tracker is an automated software system that monitors men and women’s voices on seven major Canadian news outlets in real time, using Natural Language Processing (Asr et al., 2021). The goals of the Tracker are to enhance awareness of women’s portrayal in public discourse through hard evidence and to encourage news organizations to provide a more diverse set of voices in their reporting. The Tracker has been collecting and analyzing data since October 2018. In this article, we analyze 2 years worth of news articles, a total of 612,343 articles from seven Canadian English-language mainstream news organizations.

We apply topic modelling, an unsupervised machine learning technique (Blei et al., 2003), to this data, organizing it into the top 15 most representative topics each month, and then study the distribution of women and men quoted by topic. This method allows us to discover topics across a diverse range of outlets, since we do not rely on news categories provided by the news organizations. Topic modelling can, in principle, scale to unlimited amounts of data and be deployed without much customization.

Our results show that there are clear trends in the proportion of men and women quoted depending on the topic of the article they are quoted in. Women are consistently quoted more frequently than men in the topics “Lifestyle”, “Arts and Entertainment”, and “Healthcare”, whereas men are quoted more frequently than women in the topics “Business”, “Politics”, and “Sports”. This comes as no surprise, regrettably, as the frequency of quotes seems to reflect a gendered cultural division of duties, where women are placed into the role of caregiver and nurturer and are less represented in the spheres of politics and business. This gendered division of duties and representation in media is no doubt self-reinforcing; quoting female sources more frequently in a caregiving role and quoting male sources more frequently in political and business roles enshrines women’s status as caregivers and men’s status as leaders and breadwinners (Wood, 2011; Garg et al., 2018; Ribeiro et al., 2018; Ward and Grower, 2020). Since the first step in any attempt at change is awareness, we contribute by gathering and analyzing the data in a systematic and digestible way to inform the public of these gender divides.

We would like to acknowledge that the terms we use in this paper are simplifications of a complex reality. We use the terms “women”, “men”, “female sources”, and “male sources”, implying a binary opposition that we know is far from simple. We know, at the same time, that lack of gender representation in many aspects of society is a reality. The aim of this study is to quantify that gender gap by analyzing language and the traditional associations of names with men and women.

## Materials and Methods

### Data

Data was obtained for the Gender Gap Tracker and analyzed for this related project. The Gender Gap Tracker data is scraped daily from the websites of seven major Canadian news outlets in English (Asr et al., 2021). We built scrapers from scratch for each outlet and maintain them regularly, as the sites change in structure frequently. The data is obtained through the “fair dealing” provision in Canada’s Copyright Act, which allows us to use it for research purposes. It can be made available upon request and upon signing a license agreement.

We analyzed 24 months worth of data, a total of 612,343 articles. As we see in Table 1, outlets vary in the volume of articles they publish, with CTV News being the most prolific, and Huffington Post Canada the least so. Articles range in length, roughly between 100 and 1,500 words per article, with a median length of 461 words.^2^ We provide numbers for people mentioned and people quoted. To highlight the lack of gender equality, the percentage columns in Table 1 indicate the percentage of women mentioned or quoted for each outlet and the average across outlets at the bottom.

**TABLE 1:** Data for the study, October 1, 2018–September 30, 2020. Note: “people mentioned” is the number of all persons named in all the articles per outlet. “People quoted” is the number of mentioned persons who were quoted one or more times in each article. Each “quote” is only counted once per person per article.

Outlet	Number of articles	People mentioned	Percentage women mentioned (%)	People quoted	Percentage women quoted (%)
CBC News	151,288	749,351	30.2	286,770	32.6
CTV News	158,249	565,990	28.3	240,215	29.7
Global News	86,386	353,733	25.3	133,021	30.1
HuffPost Canada	15,765	86,429	29.2	25,975	30.8
National Post	27,925	166,080	21.4	45,663	23.9
The Globe and Mail	87,121	496,142	22.1	153,881	23.2
The Toronto Star	85,609	509,851	23.1	163,255	25.1
Overall	612,343	2,927,576	25.7	1,048,780	27.9

Using the Gender Gap Tracker’s existing Natural Language Processing pipeline (Asr et al., 2021), the data is enriched with information about the people mentioned (via Named Entity Recognition) and the people quoted, whom we refer to as *sources*. For the latter, we first identify quotes (reported speech, whether direct or indirect speech) in the text and then associate a speaker with them, using coreference resolution. Finally, we look up the gender of the speakers (male, female, or other) using a mix of sources: an in-house cache of commonly quoted public figures and an API that stores the gender of people based on their first or full name. Thanks to this NLP pipeline, we obtain gender statistics (people named, people quoted, and their gender) for each article, whose aggregated form is shown in Table 1. We use these gender statistics for the topic modelling and language analyses described in this paper.

At this point, we would like to elaborate on our nomenclature. In this work, *people mentioned* refers to all unique individuals named in an article, regardless of whether or not they were quoted. These names are extracted and identified by our NLP pipeline via Named Entity Recognition. *People quoted* refers to the subset of *people mentioned* that were quoted one or more times in an article, identified via coreference resolution. The counts of people mentioned and people quoted are per article, as we do not keep track of the same individual across articles, that is, our unit of analysis is the article.

To illustrate this more clearly, consider a “set” of two articles. If the politician Chrystia Freeland is mentioned in both articles and has three quotes in one article and none in the other, this means two women were mentioned (one woman in each article) and one woman was quoted (one woman quoted three times in one article) in the set of all articles. If, however, Chrystia Freeland was quoted once in one article and once in the other, and another politician, Christine Elliott, was mentioned in the same two articles (but not quoted), two women were mentioned and two women were quoted in that set, with Chrystia Freeland counting as one woman in each article that she was quoted.

**FIGURE 1:**
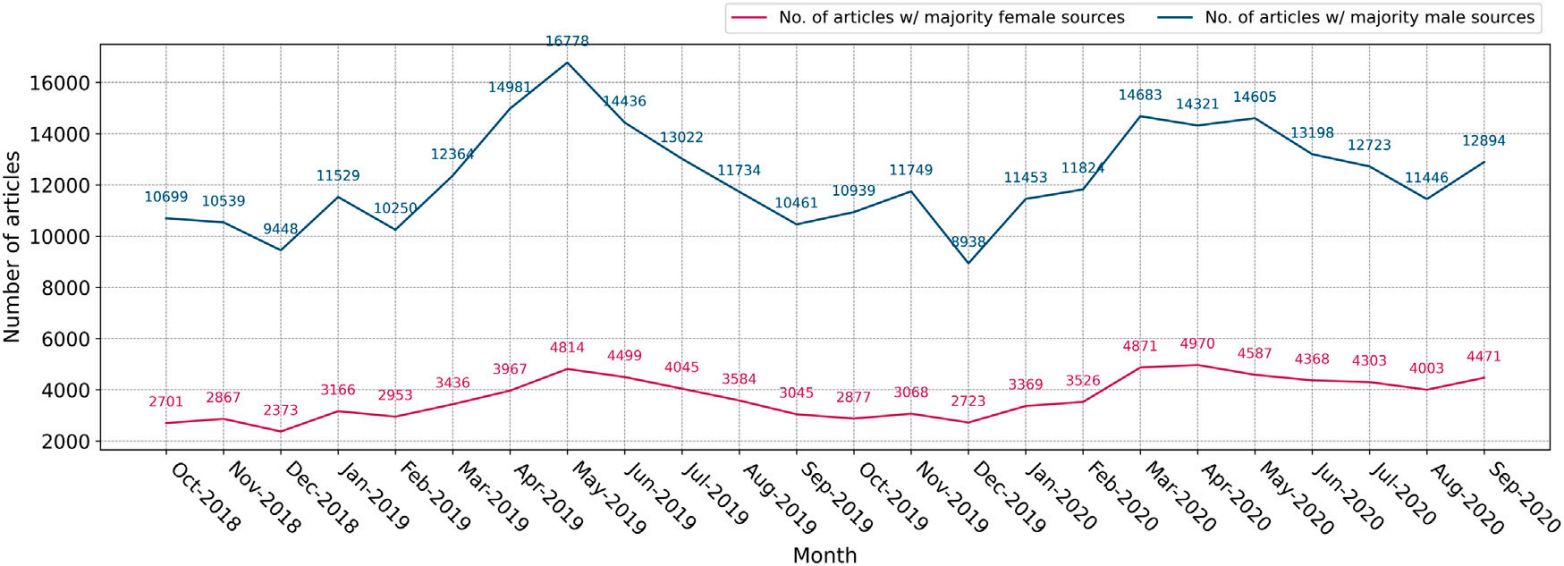
Monthly counts of articles that contain majority male/female sources.

Figure 1 shows the number of articles per month with a majority of female and male sources. The number of articles that quote more men than women is roughly 3–4 times, on average, greater than the number of articles with a majority of female sources. In other words, news outlets quote men far more often than they do women, at a ratio of about 3:1, a result that has been shown in other analyses of news media (Macharia, 2015; Shor et al., 2015; Kassova, 2020a; Kassova, 2020b). Many of these sources being quoted are frequently repeated across outlets, as well as across time periods—for example, Donald Trump, the United States President during the period of analysis, and Justin Trudeau, the Canadian Prime Minister, are the most quoted men by far, appearing as sources in a large proportion of all articles that feature politics and related topics. The fact that both curves in Figure 1 tend to rise and fall together in the same months indicates that the disparity between the raw numbers of men and women quoted is a constant feature of news coverage, and not due to volume of articles or quotes on any given time period.

Numbers alone tell a compelling story about the lack of female voices in the media, as we show in more detailed analyses of the Gender Gap Tracker data (Asr et al., 2021). Our goal in this paper is to explore how certain news topics contribute disproportionately to those percentages, and whether we can identify any systemic patterns in the language used in articles where each gender dominates, i.e., in articles with majority male or female sources.

### Topic Modelling

Topic modelling is an unsupervised machine learning technique to discover the main *topics*, or themes, in a collection of unstructured documents (Blei et al., 2003; Blei et al., 2010; Blei, 2012). A topic here refers to a cluster of words that represents a larger concept in the real world. Each document in a corpus can be imagined as consisting of multiple topics in different proportions all at once—for example, an article about a major airline procuring new aircraft may contain many words related to finance, geopolitics, or travel policies, as well as passenger trends or market events. A document can thus be composed of several topics, each represented by specific words. Topic modelling encapsulates these ideas into a mathematical framework that discovers clusters of word distributions representing overall themes within the corpus, making it a useful technique to analyze very large datasets for their content.

Topic modelling has been successfully deployed to study changes, relations, and impact in the scientific literature (Hall et al., 2008; Mimno et al., 2006) or different points of view in the news (Paul and Girju, 2010). It is particularly well-suited for organizing and classifying large numbers of documents (Eads et al., 2020; Hall et al., 2012) and has been deployed in many areas of the social sciences, e.g., for policy analysis (Isoaho et al., 2019), discourse studies (Brookes and McEnery, 2019), news media studies (Gao et al., 2012; Jacobi et al., 2016; Jia et al., 2016; Puschmann and Scheffler, 2016), or social media data (Hu et al., 2012; Nguyen and Shirai, 2015). It is a highly customizable method and results can vary depending on how parameters are set. We present a summary of the main parameters and experiments on how to optimally set them.

The mathematical goal of topic modelling is to fit a model’s parameters to the given data using heuristic rules, such that there is a maximum likelihood that the data arose from the model. Such methods are known as parametric methods, among which Latent Dirichlet Allocation (LDA) is by far the most popular. Because of LDA’s inherently non-deterministic and statistical qualities, methodological decisions involving data preprocessing, choosing the number of topics, tuning the algorithm hyperparameters, and evaluating the model’s results become all the more important (Maier et al., 2018).

LDA is framed as a Bayesian problem, in which the key issue that needs to be resolved is one of inference, i.e., computing the posterior distribution of the hidden variables. Some implementations of LDA use a sampling-based approach to compute the approximation of the true posterior. The most common sampling method used is *Gibbs sampling*, in which a Markov chain of random variables is constructed with each variable dependent on the previous ones—the limiting value of this distribution equals the true posterior. The algorithm is run on the Markov chain defined on the hidden variables for a particular corpus and a number of samples are drawn using a *Markov Chain Monte Carlo* algorithm, following which the approximate distribution is constructed from the collected samples. While sampling-based methods are guaranteed to be identical to the true posterior under limiting conditions and can produce less biased results overall, they are quite computationally expensive and do not scale as well as variational Bayes methods do, as the corpus grows in size (Blei, 2012).

The next few subsections address the methodology in our topic modelling pipeline, in addition to the experiments we conducted to arrive at each of the parameters, but see the Supplementary Material for technical details. Then, in *Language Analyses*, we detail the language analysis methodology.

### Topic Modelling Methodology

Our topic modelling pipeline uses Apache Spark’s^3^ parallelized LDA implementation^4^ via its Python interface, PySpark. The primary reasons we chose Spark are performance and *horizontal scalability*. Our news data in the Gender Gap Tracker is continually growing, with new data being added daily through the use of automated scrapers, so we do not know upfront the number of articles that may appear in any given week or month. Our database statistics tell us that, on average, we add roughly 800 to 1,500 English news articles every day, amounting to anywhere between 20,000 and 35,000 articles in any given month.

Our primary focus in this work is to build a robust, CPU and memory-efficient data and analysis pipeline that scales well. Horizontal scaling means that we can scale our computation by simply adding more distributed machines to our resource pool to handle the larger workloads. For this project, we utilize the extensive computational resources available on Simon Fraser University’s Cedar supercomputer.^5^

Due to the volume of data in the Gender Gap Tracker, we chose to avoid working with Gibbs sampling altogether. We use the online variational Bayes inference model as implemented in Apache Spark^6^ for all our experiments. In addition, we perform topic modelling via LDA 1 month at a time, organizing the data into 15 topics per month. After this automated stage, a human inspects the top 15 words per topic and assigns interpretable labels to each topic, following guidelines available in the Supplementary Material. An interactive dashboard displays topics by month.^7^

#### Preprocessing Steps

By and large, we apply topic modelling best practices in the ordering of each preprocessing step, following Maier et al. (2018). Tokenization and normalization (i.e., removing all unnecessary symbols and artifacts) are performed using regular expressions that catch only alphanumeric text while ignoring symbols and punctuation. This step is immediately followed by lowercasing, stopword removal, and lemmatization, where we reduce the inflectional forms of a word to its root form. Stopword removal is done prior to (and not after) lemmatization, primarily to reduce the initial vocabulary size, so that fewer lemma lookups are performed, thus improving performance.

**FIGURE 2:**
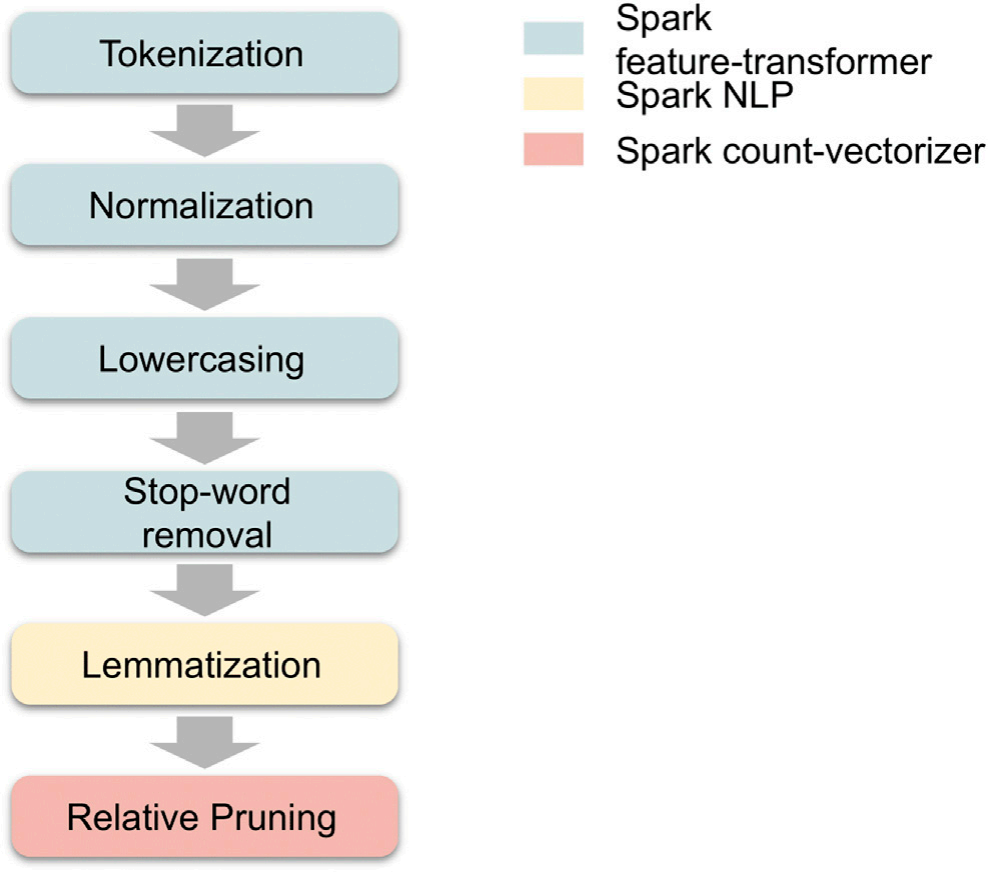
Topic model preprocessing and transformation steps.

The final preprocessing step is relative pruning, in which both very frequent and extremely rare words are stripped from the vocabulary prior to training. This is based on Zipf’s law, which, when applied to a language corpus, states that a large fraction of words in a vocabulary occur extremely infrequently (Manning and Schütze, 2003). Performing relative pruning not only reduces the size of the corpus that the model has to work with, but also helps stabilize the LDA algorithm’s stochastic inference. Figure 2 shows the sequence of preprocessing and feature transformation steps applied to the text of each news article prior to training a topic model. The boxes are coloured according to the Spark module we use for each task.

#### Stopwords

Stopwords are common words in the vocabulary that are constant across topics and do not contribute to interpretability. We formulated our list of stopwords iteratively, by beginning with a list of standard stopwords from the literature, including articles, pronouns, and prepositions. We also studied different formulations of *light verbs*, i.e., verbs that have little semantic content of their own, but instead form predicates with other expressions. Some examples are shown below (Jespersen, 1965).

*have* a rest, a read, a cry, a think

*take* a sneak, a drive, a walk, a plunge

*give* a sigh, a shout, a shiver, a pull, a ring

These verbs do not predicate fully, that is, one does not actually *take a plunge* but rather one *plunges*. However, they are not completely devoid of semantic content either: There is a clear difference between *take a bath* and *give a bath* (Butt, 2010). This makes such verbs semantically light, in a way that they are neither full nor empty in their semantic content.

From a topic modelling standpoint, light verbs do not add any value to topic interpretability. Because our news article data in the Gender Gap Tracker is unbounded in size and scope, it took multiple iterations of topic modelling to isolate specific groups of light verbs relevant to our corpus and useful for topic interpretability.

By contrast with verbs, we chose to retain a large portion of nouns during text preprocessing, due to the importance of nouns in topic interpretation. It has been shown that a combination of lemmatization and limiting the corpus to just nouns provides a coherence advantage and lower word intrusion in topic modelling (Martin and Johnson, 2015). While we do still include some very common nouns in our stopword list, these were chosen by carefully inspecting topic keywords for nouns that might cloud a human labeller’s judgment when, later, we assign labels to topics based on representative words for the topic (e.g., *people, man, woman, report, page, story*).

Our final stopword list, a sample of which is shown in Table 2, includes news media-related artifacts from the web. It also includes URL terms, social media embed terms, days of the week, and other nouns indicating time spans. Our reasoning in removing these terms is that news articles tend to describe real-world events with numerous time and date markers that do not in any way aid topic interpretability.

**TABLE 2:** Curated stopwords by category.

Category	Example words
Social media related	Post, sign, like, love, tag, star, call, group, video, photo, pic, inbox
URL and embeds	http, https, href, ref, com, cbc, ctv, src, twsrc, 5etfw
Frequent common nouns	People, man, woman, family, friend, news, report, press, page, story
Light verbs	Call, comment, continue, do, feel, give, get, take, like, make, tell, think
Time of the day/week	Morning, afternoon, evening, today, yesterday, tomorrow
Time periods	Day, week, month, year
Time zones	Edt, est, pdt, pst
Days of the week	Monday, Tuesday, Wednesday, Thursday, Friday, Saturday, Sunday
Months of the year	January, February, March, ..., October, November, December
Year	2018, 2019, 2020

#### Lemmatization

Term lemmatization is performed using a lookup process during run time, where each token is checked for presence in a dictionary of lemmas provided by the user. To do this, we turned to the spaCy NLP library,^8^ and its publicly available lemma lookup table on GitHub.^9^ spaCy is an industrial-strength NLP library for Python that is well-maintained and documented to the highest quality. The lemma list maintained by spaCy is quite extensive and contains both British and American spellings, because Canadian English tends to be based on both British and American English (Dollinger, 2019). We reformat this spaCy lemma list into a format that is compatible with the Spark NLP library and apply it in our preprocessing workflow.

### Hyperparameter Tuning

In this section, we discuss the key LDA hyperparameters we tuned to achieve our best results. Some additional modelling experiments are discussed in the Supplementary Material. Each hyperparameter is varied individually, and the quality of the result is measured using a combination of model perplexity and human judgment. In Spark, model perplexity is represented by the logarithm of the upper bound on the negative log-likelihood of tokens, divided by the number of tokens in the corpus after relative pruning. A lower perplexity means that the model achieves a better-quality fit to the given data. We define a “good” topic distribution as one that has low perplexity and is highly interpretable, with good topic separation and limited word intrusion (i.e., not too many occurrences of the same words across many topics).

#### Maximum Iterations

The online variational Bayes algorithm was observed to converge quite rapidly (within 100–150 iterations). By contrast, we found that expectation maximization can take upwards of 200 iterations to converge. This makes sense, since the online algorithm is known to speed up convergence by using stochastic optimization and is less prone to getting stuck in local optima. We did not notice much of a difference in terms of perplexity as well as topic interpretability when running the online algorithm for 150 iterations and above. Minor differences in topic keywords were noticed for specific months’ results when we used just 100 iterations. As a result, we fix the maximum number of iterations to 150 for all our training runs, regardless of the size of the data involved each month.

#### Relative Pruning

Three relative pruning hyperparameters are used during data preprocessing: maximum document frequency, minimum document frequency, and minimum term frequency. Controlling these values prunes, i.e., removes, those terms that occur both very frequently and very rarely from the vocabulary. As a result, these hyperparameters must be chosen carefully, so as to not hinder topic interpretability or miss larger themes in the data.

We found that a maximum document frequency of 80% (maxDF=0.8), i.e., pruning tokens that occur in more than 80% of all documents in the corpus, worked well over multiple experiments. This value resulted in minimal loss of information while retaining a good deal of topic interpretability and separation. Too high a value (maxDF>0.9) resulted in a lot of words repeating across topics, increasing word intrusion (and hence perplexity), while reducing topic interpretability.

The minimum document frequency hyperparameter (minDF) has a significant impact on tokens that appear very rarely in the data. When setting minDF=0.05, i.e., pruning those tokens that occur in fewer than 5% of all documents in the corpus, we observed to our surprise that there was a significant drop in model perplexity. This was accompanied by a significant loss of topic interpretability, with multiple topics repeating very similar word distributions. Upon closer inspection of the topic keywords, we saw that by removing up to 5% of the least frequent tokens in the data, we were removing a large portion of the useful nouns and adjectives that aided topic interpretability. As a result, the model’s perplexity dropped significantly, due to a smaller vocabulary overall and the fact that the model was overfitting to a much-diminished distribution. In the end, we obtained our best results using a minimum document frequency of 2% (minDF=0.02). This confirms findings from previous work (Chang et al., 2009), suggesting that a model with a lower perplexity is not necessarily a better one.

The minimum term frequency hyperparameter prunes terms that occur fewer than a specified number of times *within* a given document. Unlike the document frequency hyperparameters (which are specified as percentages relative to the whole corpus), the minimum term frequency is specified as a positive integer. A minimum term frequency of 1 means that no terms are pruned from a document, whereas setting minTF=2 means that all terms that occur less than twice (i.e., just once) within a document are pruned. Our experiments with minTF>1 once again resulted in the model having a lower perplexity—however, this is primarily because pruning terms that occur once per document causes a huge reduction in the number of available terms (many terms in a document occur just once). Recall that, at this point, we have already removed stopwords, so most of the words left in the document have meaningful content. This loss of information is undesirable, and results in niche, overly specific topics being discovered in the data. Thus, we fall back to keeping the minimum term frequency as 1 (minTF=1) so that no terms within a document are pruned.

#### Vocabulary Size

Setting a finite vocabulary size limits the dimensionality of the random matrices that are solved for during LDA (i.e., θ and β) based on the relative frequencies of tokens in the corpus. In principle, the LDA algorithm can be run on datasets that are unbounded in size, and as a result, drawing Dirichlet samples from larger and larger distributions of words adds more noise to the inference process. Our survey from existing literature showed that a typical vocabulary size used in LDA ranges from 5,000 to 10,000, regardless of the size of the corpus. We tested the impact of three different vocabulary sizes in training models over one month’s worth of data (vocabSize=5000 | 10000 | 20000).

Our results showed that a larger vocabulary size has close to no impact on model perplexity and a negligible impact on interpretability. The increased sample size from which Dirichlet distributions are drawn resulted in slightly poorer topic separation and slower convergence toward the optimum. Because of its relative lack of impact on model quality, we choose to limit the vocabulary size in our models to 5,000.

#### Best Hyperparameters Obtained After Fine-Tuning

Our fine-tuned topic modelling workflow in production involves a semi-automated process that trains individual topic models on one month’s worth of data at a time. We use the best combination of model hyperparameters (shown in Table 3), and a fixed random seed of 1, based on our experiments shown in this section. Our data preprocessing methodology incorporates the curated list of stopwords shown earlier in this section, as well as a custom lemma lookup table based on the one from the spaCy NLP library.

**TABLE 3:** Best LDA hyperparameters obtained for a monthly analysis of news data.

Hyperparameter	Description	Value
K	Number of topics	15
maxIter	Maximum iterations	150
vocabSize	Vocabulary size	5,000
minTF	Minimum term frequency	1
minDF	Minimum document frequency	2%
maxDF	Maximum document frequency	80%

### Language Analyses

To gain an understanding of how certain topics present and discuss the topics and the people quoted, a deeper linguistic analysis is required. Because we divide our news article content into two separate corpora based on which gender is most quoted (see below), our scenario is well-suited to *corpus studies*, a set of techniques that are known to “help deconstruct hidden meanings and the asymmetrical ways people are represented in the press” (Caldas-Coulthard and Moon, 2010). Corpus analyses have proven fruitful in uncovering linguistic aspects of gender and gender representation (Murphy, 2010; Baker, 2014; Motschenbacher, 2015; Kemble, 2020).

For the language analyses, we rely on two key concepts from corpus linguistics, keyness and dependency bigrams. Keyness aims to identify large differences between the frequency of word-forms in two or more corpora (Gabrielatos, 2018; Pojanapunya and Todd, 2018). Keyness analysis introduced the technical concept of a “key word”, a word that occurs in a text more often than we would expect it to occur by chance alone. By comparing the words that are more representative of one corpus than the other, we can explore the differences in themes between two corpora.

A relatively more advanced form of corpus analysis, dependency bigrams identify verb-direct object combinations and their frequencies within a corpus (Sidorov et al., 2013; Mertz et al., 2014). To extract these bigrams, a dependency parser identifies words that are syntactically connected by a head-dependent relationship. For example, in the sentence *The player* ***kicked*** *the* ***ball***, the main verb *kicked* is connected to the noun *ball* via a direct object relationship, wherein *kicked* is the head and *ball* is the dependent. The dependency bigram returned using this process is “ball_kick”.

Dependency bigrams reveal linguistic aspects of the male and female corpora, unlike keyness, which mainly highlights textual or lexical differences. We obtained much more useful results with dependency bigrams rather than the more standard corpus analysis methods such as collocation or n-gram frequencies (McEnery and Hardie, 2011). This is because the dependency bigrams capture syntactic and semantic relationships, rather than just bags of words from a text (unigrams) or linear relationships (regular bigrams). The next section details how we created gender-prominent corpora for five main topics and the results obtained in these language analyses.

## Results

### Monthly Gender Prominence for Recurring Topics

**FIGURE 3:**
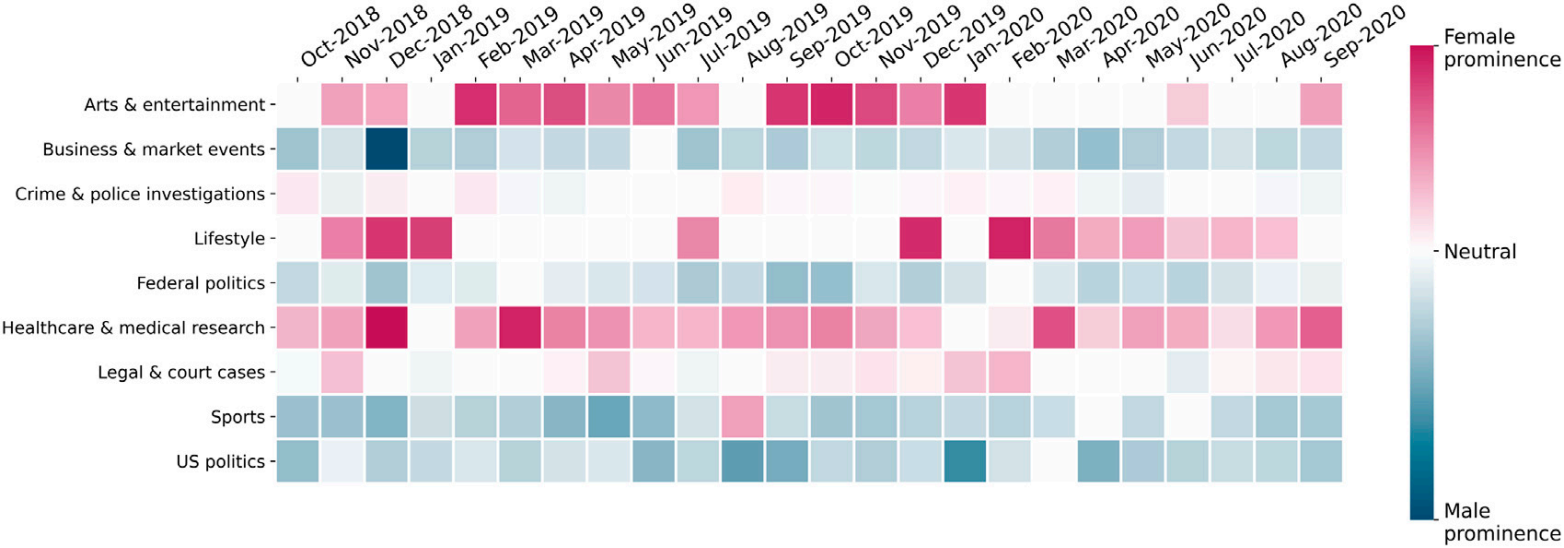
Monthly topic gender prominence for nine recurring topics (average over all outlets).

We examined nine topics that feature regularly in most months between October 2018 and September 2020. The topics cover a broad range of domains typically seen in the news, as seen on the vertical axis of Figure 3. From our dashboard (https://gendergaptracker.research.sfu.ca/apps/topicmodel), we obtain per-month, per-outlet mean topic gender prominence measures for each of these topics over the 2-year period. We define gender prominence as the difference in mean topic weights between the female and male corpus or dataset for a given topic. A topic is categorized as having male prominence if the mean topic weights from the male corpus are greater than those from the female corpus. See the last section in the Supplementary Material for more details on how we define male and female prominence and how we separate topics into male and female corpora.

The values for prominence range from positive (red in Figure 3), indicating female prominence for that topic, to negative (blue), indicating male prominence. We tabulate these numbers per outlet and topic for the entire duration, and then calculate the mean gender prominence for each topic over all outlets. The resulting table is plotted as a time-series heat map, as shown in Figure 3. The white (neutral) squares in the heat map indicate that a given topic did not appear at all in that month (or, in rare cases, that there is perfect gender parity in sources for that topic and month).

Figure 3 shows that there are topics that clearly exhibit male or female prominence over extended periods of time. This indicates that, for specific topics, news outlets tend to consistently feature either men’s or women’s voices more frequently, resulting in majority male or female sources quoted in a large fraction of the articles for that topic, regardless of the outlet. For example, the topic “Arts and entertainment”, which primarily discusses news about public events, art exhibitions, films, television, and celebrities, tends to display a high topic intensity in the female corpus. We see the topic is not as widely covered in February–May 2020, likely because, in the first few months of the COVID-19 pandemic, most live arts events were cancelled.

Other topics that display strong female prominence over time include “Lifestyle” (which contains keywords related to families, home/holiday activities and individual/personal experiences), “Healthcare and medical research”, and “Legal and court cases”. The primary reason for the “Lifestyle” topic being female-prominent is that it regularly features mothers involved in childcare, women reliving their past experiences, and female experts offering personal care advice. The “Legal and court cases” topic tends to feature more women mainly due to cases that pertain to sexual assault or women’s rights, featuring both victims and experts who are women. The “Healthcare and medical research” topic is particularly interesting, because, in Canada, a sizable proportion of healthcare experts and Public Health Officers happen to be women (Fitzpatrick, 2020). This leads to the healthcare topic regularly featuring quite strongly in the female corpus, with a range of women’s health issues being covered and prominent expert women being quoted.

On the other hand, the “Business and market events”, “Sports”, “Federal politics”, and “United States politics” topics are almost entirely male-prominent throughout the period studied. The “Business” topic typically includes keywords pertaining to investments, analysts’ predictions on the stock market, and in-depth discussions on company futures, interest rates, and profits. Historically, these areas tend to be dominated by men, partly explaining why the topic intensity for “Business” is always much lower for the female corpus. Similarly, the “Sports”, “Federal politics”, and “United States politics” topics feature discourse that generously quotes male athletes, coaches, and politicians, who are not only more in numbers in society, but are also quoted much more often than women.

In general, the heat map in Figure 3 highlights aspects of societal bias that we know exist today. It also seems to uphold our topic modelling methodology, as it confirms the intuitions that we had when we started this project. We explore these trends for specific topics in the next sections, focusing on the language in each of the topics.

### Language Analysis for Recurring Topics

Using the results from LDA, we characterize each individual news article in our corpus for a particular month as belonging to a *distribution over topics*. Mathematically, this means that the topic modelling process returns a one-dimensional vector of length 15 (we only model a maximum of 15 topics each month) for each document, where each component of the vector, which we call a *topic weight*, represents how strongly or weakly that topic’s keywords are associated with that document. To begin the combined topic and language analyses, we identify a topic of interest that exhibits strongly male or female gender prominence for a particular month. We then query that month’s data from our database and sort in descending order of topic weights for that topic. Sorting the articles in this order places all articles that are strongly related to that topic’s keywords on top. The full corpus, in sorted order for a particular topic, is then split into two corpora, each with male-majority and female-majority sources (see the Supplementary Material for more details).

Once we have the two corpora, we extract the full text for the top 200 articles in either corpus (see below for some exceptions in months when an issue seems to be covered across multiple topics). We chose 200 articles for empirical reasons—we observed that in most cases, the maximum topic weights for each article rapidly dropped to less than 0.5 after a few hundred samples. Following these steps, we perform keyness analysis and extract dependency bigrams on five topics: “Sports”, “Business and market events”, “Lifestyle”, “Healthcare and medical research”, and “Crimes and sexual assault”. Below we discuss our analyses of these five topics for certain months.

#### Sports

The first topic whose language we study in detail is “Sports”, for the months of June, July, and August 2019. August 2019 was an outlier because it was the only month for which the “Sports” topic demonstrated an overall female prominence. August 2019 was also particularly interesting because “Sports” was the strongest topic in both the female and male corpora. To better understand these trends, we performed keyness and dependency bigram analyses for the top articles from each of these 3 months. The keyness results uncover lexical differences between the female and male corpora, whereas the dependency bigrams show syntactic (i.e., structural) differences based on subject-direct object relationships, that is, events and relationships that the articles capture.

**FIGURE 4:**
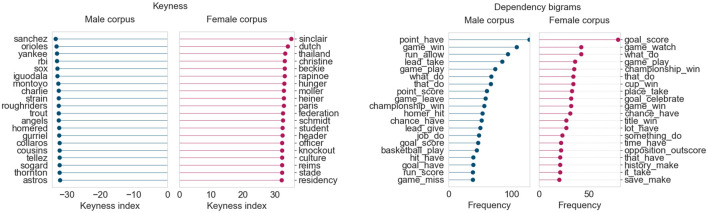
Corpus analysis results for top 400 articles from “Sports” (June 2019).

Figure 4 displays the results for June 2019. For this month, we obtained two topics for sports: one for baseball/football, and another for basketball due to the Toronto Raptors becoming NBA champions. As a result, we consider the top 200 articles from both sports topics for this month’s analysis, giving us 400 articles in each corpus for this month.

From the keyness index plot, we can see that the female corpus contains numerous terms pertaining to the FIFA women’s world cup held in France that month. The names of Canadian and American soccer players and those of their female coaches appear, as do other terms related to women’s soccer federations. The male corpus, on the other hand, contains terms largely related to baseball (*Yankees*, *Red Sox*) and teams from the Canadian Football League (*Lions*, *Roughriders*). The dependency bigram plots indicate that there are differences in language across the female and male corpora, based on frequency counts of the most common bigrams. (The dependency bigrams in the plots should be read right-to-left, that is, “game_win” is part of a sentence involving *win a/the game*, and “lead_take” refers to taking the lead in a game.) The male corpus contains much higher occurrences of general verbs describing events in baseball/football games, such as *hit homer, score goal, allow run,* and *give lead*. The female corpus seems much more focused on winning, celebrating, and breaking records (although *score goal* is the highest-ranking bigram in terms of raw frequency).

**FIGURE 5:**
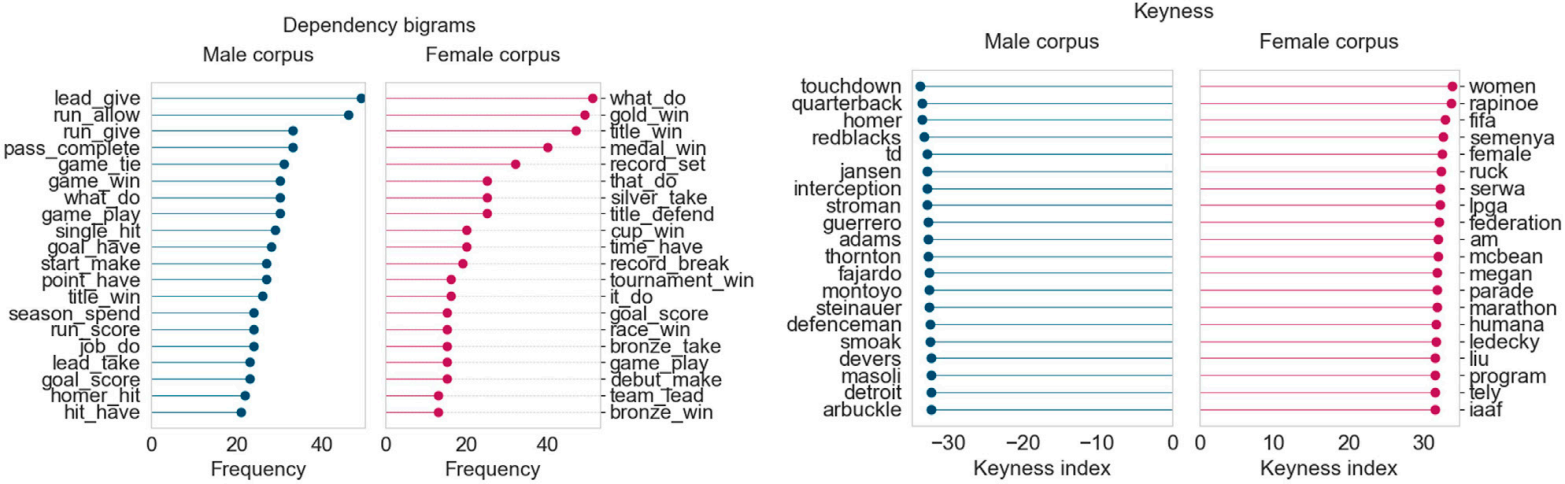
Corpus analysis results for top 200 articles from “Sports” (July 2019).

July 2019 (Figure 5) follows a similar trend to June 2019. The female corpus continues to include terms related to the FIFA women’s world cup, with some added keywords and person names related to summer athletics and golf (*Semenya*, *Rapinoe*, *Liu*). The male corpus continues to highlight terms and player/coach names from baseball (*Blue Jays*) and the CFL (*Redblacks*). The dependency bigram plots also reveal comparable results, with the female corpus using more vocabulary related to winning cups/medals and setting/breaking records. On the other hand, the male corpus much more frequently highlights terms that describe specific aspects of games, such as giving the opponents the lead or allowing them a run.

**FIGURE 6:**
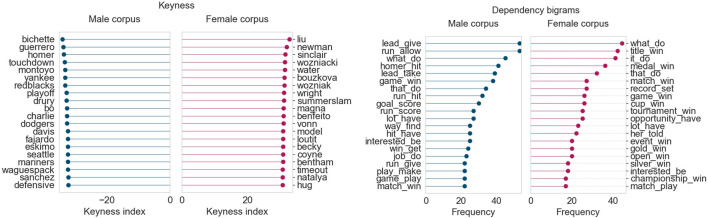
Corpus analysis results for top 200 articles from “Sports” (August 2019).

Finally, in August 2019, shown in Figure 6, the keywords point to a switch in focus towards the Wimbledon tennis tournament and summer athletics. The male corpus continues to focus heavily on baseball and the CFL season and their personnel. Just as in the prior two months, the female corpus once again shows a high frequency of bigrams related to winning matches, titles, or cups, and setting records.

This analysis of the top “Sports” articles across three summer months in 2019 shows that articles that feature women’s voices tend to focus more heavily on achievements and records (*win cup* and *break record*), whereas articles that feature men’s sports focus on the finer details of each game. Perhaps authors that write about women’s sport highlight women’s achievements and success stories because of the limited time and space they have, compared to the coverage given to men’s sports. After all, the baseball and football seasons in Canada are each several months long, whereas events like the FIFA world cup and the Wimbledon tournament occur in the span of just 1 month. It does seem that, when it comes to sports, men just need to play to feature in the news; women need to win.

Sports does not have to be a male-dominated arena in the news. In sports that have well-known female role models (Bianca Andreescu for tennis, Christine Sinclair and Megan Rapinoe for soccer, and Michelle Liu for golf), we see considerable amounts of coverage in the female corpus during certain times of the year. Sustaining a high level of coverage, however, seems to require more women role models across a wider range of sports, as well as longer seasons over which women’s sports are regularly featured. Since the COVID-19 pandemic in early 2020, our topic model gender prominence results show that women’s sport has once again taken a back seat in the news.

The underrepresentation of women in sports news has been amply documented, showing that coverage has not kept pace with the increase of women’s participation in sports (Duncan et al., 2005; Cooky et al., 2013). However, research on sports reporting shows that this traditional gendering can be countered, especially when more female reporters are assigned to cover sports events (Kian and Hardin, 2009). We have found in our previous work that female writers are more likely to quote women (Asr et al., 2021), but more extensive analyses suggest that there is no relationship between the proportion of women producing the news and the proportion of women featured in the news (Kassova, 2020b). It is possible that the culture in newsrooms is male dominated, thus resulting in professional identity being stronger than gender identity (Hanitzsch and Hanusch, 2012; Kassova, 2020b).

#### Business and Market Events

We consider 2 months, October and December 2018, for which this topic exhibits a particularly strong male prominence. The top 200 articles with the strongest topic intensities in each month were chosen for comparison.

**FIGURE 7:**
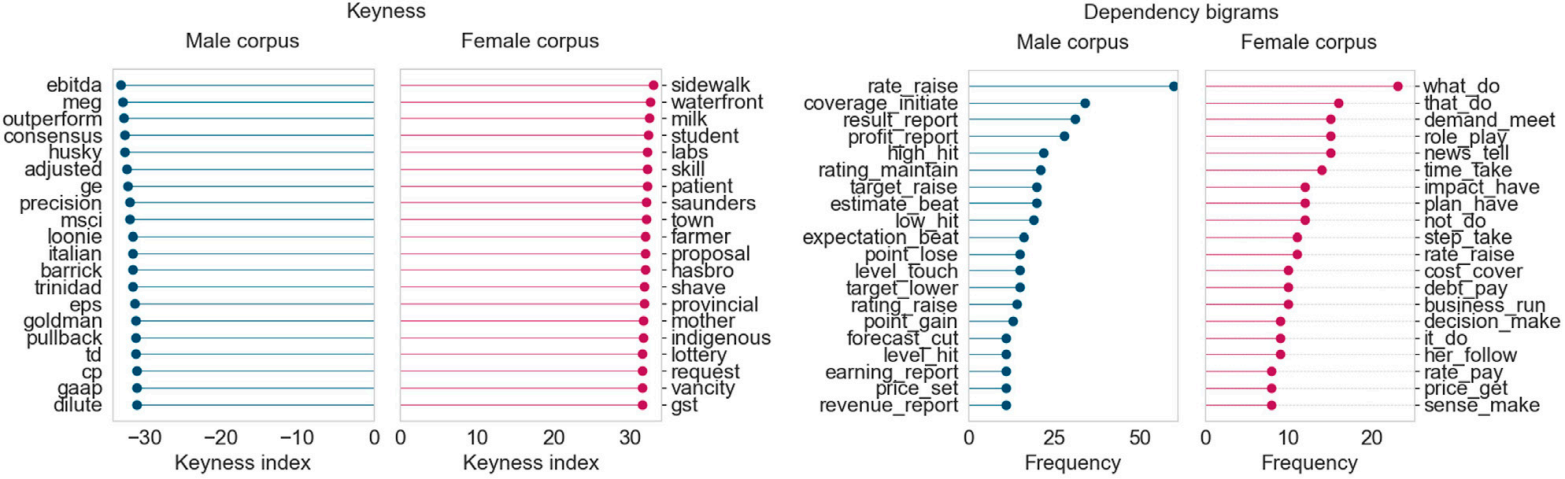
Corpus analysis results for top 200 articles from “Business” (October 2018).

In October 2018 (Figure 7), in the female corpus we see words related to small transactions and shopping (*milk*, *shave*) and small/local businesses (*town*, *mother*). The dependency analysis gives us expressions like *have impact*, *cover cost*, *pay debt*, and *run business*. The male corpus contains terms related to larger financial instruments, stocks, trading, and company performance metrics. The male corpus also frequently features verbs pertaining to high-level business policy and decision-making (*raise rate*, *maintain rating*, and *report profit* bigrams).

**FIGURE 8:**
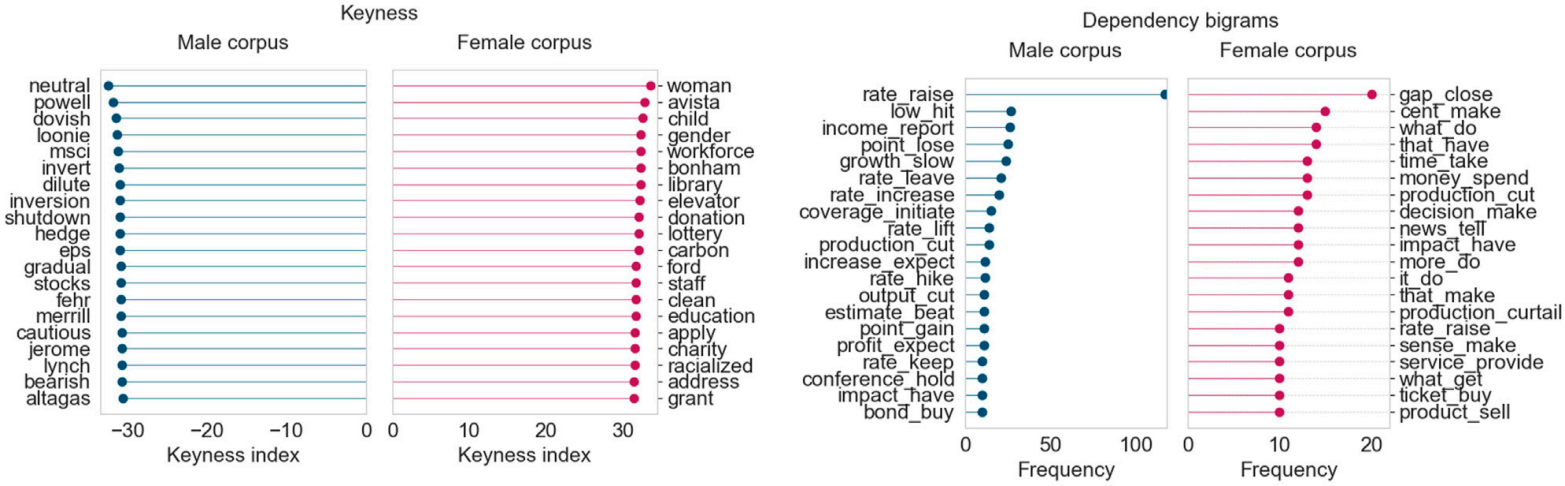
Corpus analysis results for top 200 articles from “Business” (December 2018).

December 2018, the month for which the “Business” topic exhibits the strongest male prominence in all our results, showcases a similar disparity in the kind of language used across both corpora, as seen in Figure 8. Keyness analysis shows that the female corpus uses keywords pertaining to women and minorities in the workplace, along the lines of *gender*, *workforce*, *child*, and *racialized*. The male corpus, as always, contains terms very specific to financial and business operations (*stock*, *hedge*, *dilute*, *bearish*). Inspecting the dependency bigrams, we can see that the female corpus highlights terms such as *cut production* and *make decision*—this was primarily due to then-Alberta Premier Rachel Notley’s numerous comments on the oil industry’s production cuts. Other terms such as *close gap*, *spend money*, and *provide service* clearly point to some level of gender disparity between the two corpora.

We find that the “Business and market events” topic regularly exhibits the largest disparity between the male and female corpora, in terms of language used, for all months of data studied. It seems that women’s voices are rarely featured in articles that cover business, especially big business, finance, and the stock market. The kind of language used (as seen in the dependency bigrams) shows that the focus of articles that quote women tends to be primarily small/local businesses and the challenges of working at or running a (small) business. It is also important to note that a large portion of articles in this topic were published by *The Globe and Mail*, which we know quotes, on average, the smallest proportion of women.

#### Lifestyle

**FIGURE 9:**
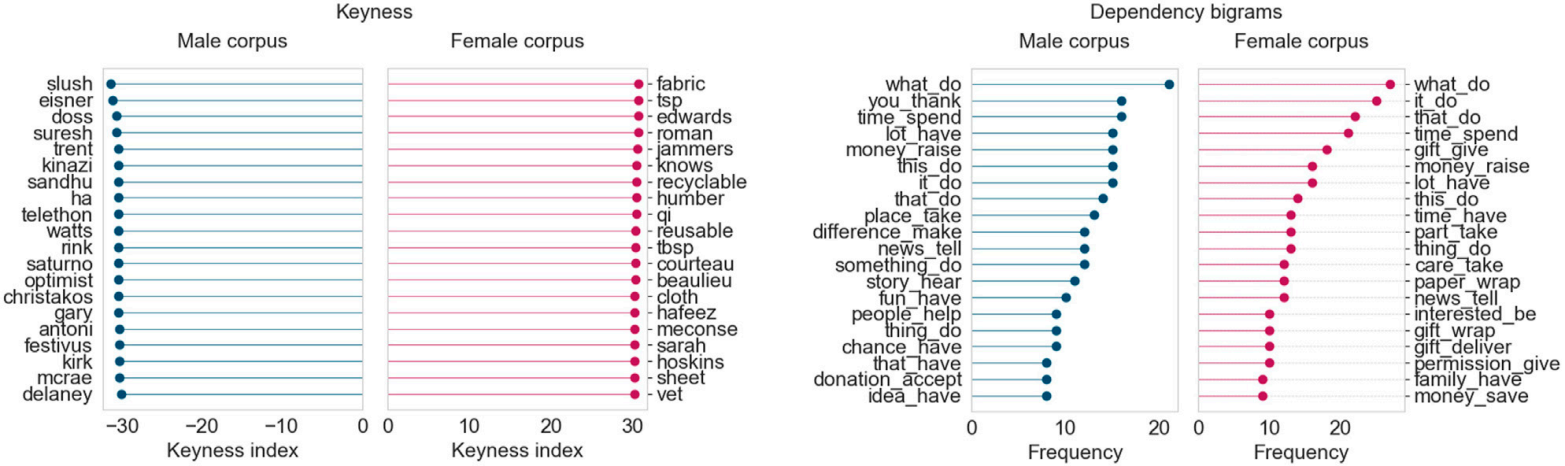
Corpus analysis results for top 200 articles from “Lifestyle” (December 2019).

The “Lifestyle” topic, which covers content related to families, personal experiences, holidays, and shopping, exhibits strong female prominence for all months in which it appears. Figure 9 shows the results from December 2019, which, together with February 2020, had particularly strong female prominence in this topic.

Keywords from the female corpus for December 2019 highlight a range of lifestyle topics, from food recipes (*tbsp*, *tsp*) to clothing and fabrics. Interestingly, not as many women’s names are seen for this month’s keywords as for other topics in other months. The male corpus keywords include several names of people, including Suresh Doss, a Toronto-based food writer. The dependency bigrams for both the male and female corpora focus mainly on the festive season, as well as acts of gift-giving and donating to charities.

We find that the “Lifestyle” topic, unlike other topics, tends to name regular people who share their life experiences with the media—this can lead to the drowning out of other keywords such as common nouns or adjectives in the keyness results. As a result, keyness is slightly less useful in interpreting textual disparities across the corpora for this topic. On occasion, interesting terms (such as *parentese*, the speaking style used by parents of young children, also sometimes referred to as *motherese*) emerge, mainly because multiple outlets tend to write about the same topic, typically quoting the same people.

The most interesting question here is one of cause and effect, that is, whether specific keywords emerge in the female corpus because they are discussed by expert women, or whether women are approached to discuss specific topics. We see a correlation between keywords and the gender of those quoted; unfortunately, we do not have any evidence of causation or the possible direction of that causation.

Our results are consistent with other topic-based analyses in large-scale corpora. For instance, Devinney et al. (2020) found that an explicitly gendered feminine topic (defined as having a higher proportion of female pronouns and words) was more strongly associated with the private sphere and relationships than a male-dominated topic.

#### Healthcare and Medical Research

In the Gender Gap Tracker dashboard, we observed during the period February–May 2020, at the initial peak of the COVID-19 global pandemic, that the proportion of women quoted across all outlets increased by 3–4%, compared to pre-COVID levels. To study this in greater detail, we first compared the keyness and bigram frequency results for the month of March 2019, followed by a similar analysis for March 2020, when COVID-19 began spreading in North America.

**FIGURE 10:**
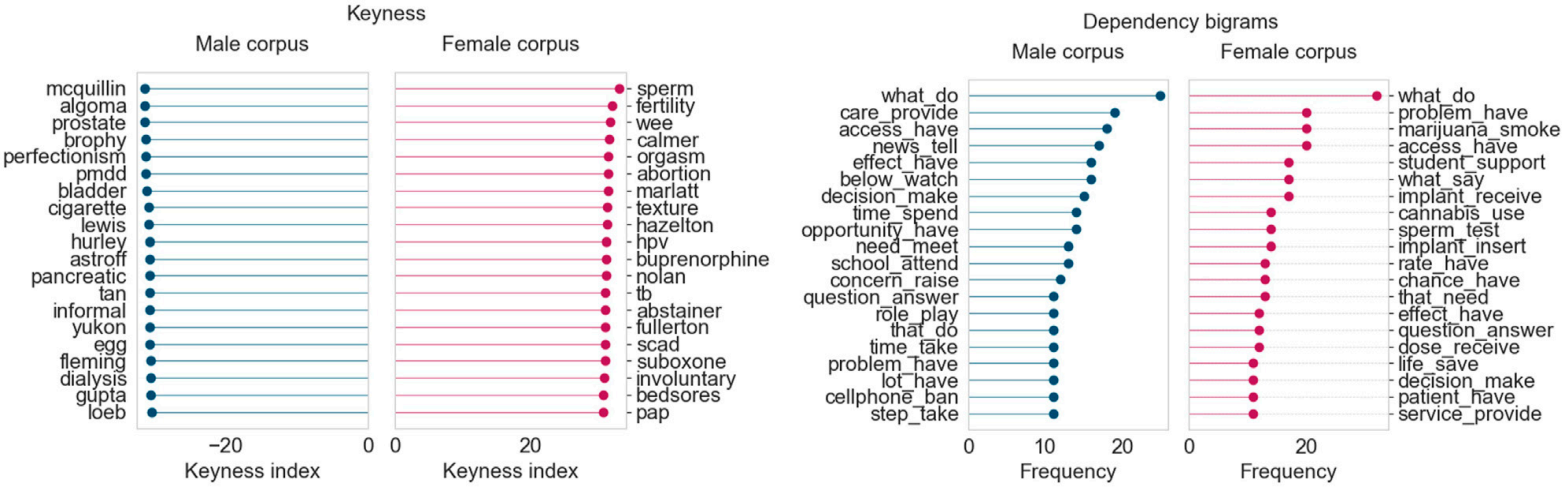
Corpus analysis results for top 200 articles from “Healthcare and medical research” (March 2019).

The female corpus for March 2019 (Figure 10) contains terms related to women’s fertility and their sexual/overall health (*sperm*, *hpv*, *abortion*), while the male corpus shows a similar focus toward terms pertaining to men’s health (*prostate, bladder, pancreatic*). Interestingly, the bigrams from the female corpus seem to point to an additional emphasis on the effects of women’s personal choices on fertility and pregnancy (*smoke marijuana*, *use cannabis*, *insert implant*). The male corpus bigrams, in this case, do not seem to have a particular focus other than the availability and effects of various treatments in the healthcare domain (*provide care*, *make decision*, *take step*).

**FIGURE 11:**
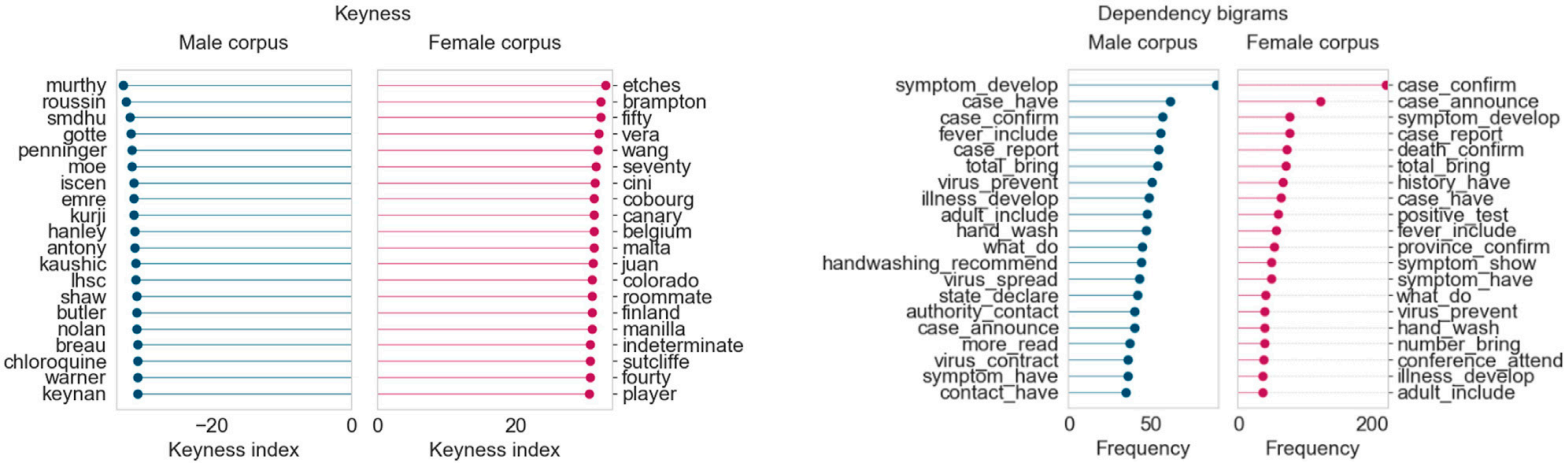
Corpus results for top 400 articles from four “Healthcare and COVID-19” topics (March 2020).

Once the COVID-19 pandemic began spreading in Canada in March 2020, there was a stark shift in healthcare coverage. Because of the seriousness of the pandemic, there were multiple COVID-related topics in March. We focus our attention on four topics that were clearly female dominant throughout this period (“COVID-19: Testing and tracing”, “COVID-19: Cases, deaths and spread”, “COVID-19: Community impact and closures”, and “COVID-19: Provincial updates and case counts”). The text of the top 100 articles for each topic (ordered by topic intensity) was collected and separated into male and female corpora, just as before. This gave us 400 articles in either corpus to analyze for their language used during this month. Keywords and dependency bigram for this corpus are shown in Figure 11.

Some of the keywords in the female corpus are names of women who are medical experts (Dr. Vera Etches and Dr. Penny Sutcliffe) or place names related to the spread of COVID-19 in various regions of Canada and the world. Similarly, the male corpus also names prominent medical researchers and scientists (Dr. Srinivas Murthy and Dr. Brent Roussin). The dependency bigrams show a very similar distribution across both male and female corpora, with terms pertaining to symptoms, case counts, and public health guidelines. Notably, the frequency count of the bigram *confirm case* is significantly higher (>200) in the female corpus than the male corpus. This makes sense, considering that, for the most part, the Provincial Health Officers confirming the numbers happened to be women.

In general, we observe that Canadian media outlets tend to provide a good degree of coverage to women’s healthcare and health issues throughout the year. This is helped by the fact that a relatively large proportion of healthcare experts and medical officers in Canada are women, which explains the high female prominence of the topic in most months of the year. The most notable of these is Dr. Bonnie Henry, the Chief Provincial Health Officer of British Columbia, who is the top quoted woman by far throughout the spring/summer of 2020, due to the COVID-19 pandemic. This leads to the conclusion that having women in prominent positions increases their chances of being quoted by the media, though this is, naturally, beyond the control of news organizations and journalists.

#### Crimes and Sexual Assault

In exploring our results, we observed that topics pertaining to women’s rights and sexual assault emerged around February and March, in both 2019 and 2020. We explore these topics for these periods using corpus studies, as shown below.

In February 2020, terms pertaining to women’s and trans rights appear in the female corpus (*hijab, trans, hormone*). The issue of the gender pay gap in sports was also a major one during this period, shown through keywords such as *fifa* and *cup*. The male corpus keywords primarily feature names, presumably of men involved in crimes and their victims. Some key dependency bigrams from the female corpus include *protect athlete, involve victim,* and *take action*, indicating that coverage was focused on the action taken toward women’s justice. The male corpus highlights bigrams related to the crimes themselves, convictions, and legal matters.

**FIGURE 12:**
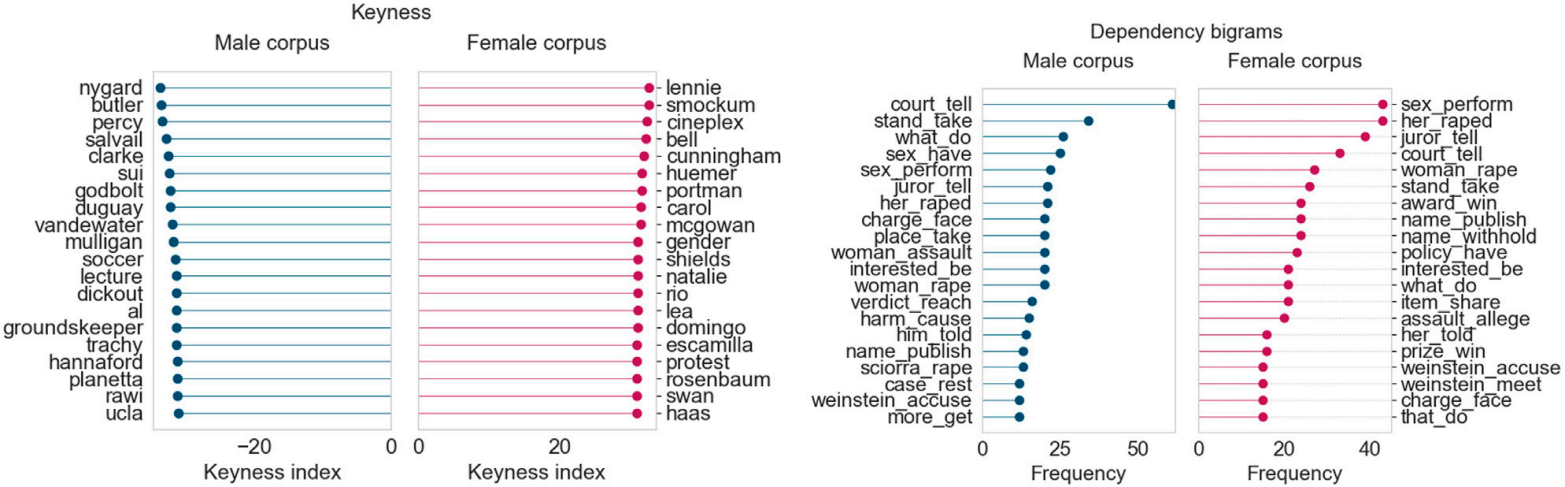
Corpus analysis results for top 200 articles from “Crimes and sexual assault” (February 2020).

In February 2020, as seen in Figure 12, several keywords in the female corpus relate to the Harvey Weinstein sexual crime convictions, as well as other crimes against women. Several survivor names appear in the keywords (*mcgowan, rosenbaum, cunningham*). The male corpus keywords once again consist of names of the perpetrators or victims involved. In general, the language used in the female corpus (as seen in the bigrams) tends to focus on the victim’s accusations and details of the crimes, and on the convictions, defense, and accounts of the victims in the male corpus.

It is quite interesting that topics around women’s rights (encompassing themes from sexual assault, consent, #MeToo, and gender equality) tend to emerge at the same time each year, around February and March, which is the time when International Women’s Day is celebrated. Analyzing the language used, we also find it heartening that strong action verbs (*take stand, tell story*, *have right*) are used quite frequently in the female corpora, highlighting the role the media plays in spreading awareness about women’s issues and rights in Canada.

## Discussion

### Key Findings

Topic modelling of a large dataset of news articles reveals persistent gender inequality in who is quoted depending on the article’s topic distribution. We found that, out of the main topics that we consistently see month-to-month in our 2-year study, women are quoted more frequently when the topic relates to lifestyle, healthcare and medical research, and crimes and sexual assault. Men, on the other hand, see their voices reflected more frequently when the topic relates to politics, business and markets, and sports. In other words, men dominate in front-page news, “the more consequential spheres of public life” (Kassova, 2020b, p. 89) and women in what can be described as lower-profile news genres.

Analyses of the language used in articles with a majority of men or women as sources reveal that business articles show a marked disparity in the kind of language used. Business articles that quote men heavily tend to highlight aspects of big business and the stock market, whereas articles that quote more women than men tend to focus on small/local businesses and shopping transactions. Sports, a male-dominated area of the news, also shows interesting disparities. When women are prominent in sports, the narrative is about winning, rather than just about describing specific games or protagonists of those games.

The results presented here confirm findings in our previous work, which shows that, on average and regardless of topic, men are quoted about 70% of the time in Canadian news stories (Asr et al., 2021). While the findings are, sadly, not surprising, they paint a clearer picture than has been available so far. Our analyses show a consistent gender divide by topic, highlighted not only by the relative proportion of men and women quoted by topic, but also enriched through analyses of keywords and dependency bigrams.

One potential objection to our work is that perhaps the relative proportions of men and women in the different topics simply mirror the distribution of men and women in those spheres, and that this might not point to a bias in the media at all. While this is a valid point, it is quite difficult to address quantitatively, as it is not easy to determine what that real-world baseline (in terms of men and women in each sphere) is. Almost every sport has a men’s and women’s division. But it’s often the men’s that is more lucrative and/or prominent. In that case, setting the baseline to the number of prominent or professional men athletes, would, in our view, simply reinforce the existing bias. This is why we assume a 50% baseline, that is, the approximate proportion of women in the world, in order to highlight the existing gap in women’s representation in the media.

From a methodological point of view, we showed how an online variational Bayes algorithm, in conjunction with an adaptive optimization policy to obtain asymmetric Dirichlet parameters *α* and *β*, reliably produces topics that align well with events from the real world, while also effectively scaling to large datasets. In contrast to prior work that recommends removing stopwords as a post-processing step (Schofield et al., 2017) to aid in topic interpretability, we found that applying a hand-curated stopword list during preprocessing and using linguistic concepts such as light verbs consistently produced semantically coherent topic keywords that could easily be labeled by a human. Our findings agree with existing work on qualitative evaluation of topic models (Chang et al., 2009), which suggests that traditional measures of topic quality based on predictive likelihood (such as perplexity) are anti-correlated with semantic coherence, as determined by a human.

The topic modelling framework we present is combined with a novel visualization technique that superimposes gender statistics of people quoted with the topic breakdown for the corpus, which, to our knowledge, has not been done prior to this work. The dynamic nature of the approach captures prominent news events monthly, allowing us to visualize ongoing trends, while at the same time identifying interesting subsets of our news corpus that show marked gender disparities.

### Limitations of Our Approach

Our topic modelling pipeline was built to scale to huge amounts of data, so some compromises had to be made to maintain topic interpretability, robustness, and performance. We elaborate on three potential limitations, regarding the use of bigrams, evaluation metrics, and random seeds.

First, one of the important decisions we made was to break up each article into unigrams, that is, individual words, rather than sequences of words of any length (n-grams). During feature transformation, a common approach is to tokenize the corpus into bigrams (Wallach, 2006; Wang et al., 2007; Lau et al., 2013). Words that regularly occur together, such as “United” and “States”, should, ideally, be tokenized as “united_states”, by counting how often these terms occur together in the corpus. We opt against counting bigrams in our workflow mainly because of concerns with memory requirements due to the size of data. In this study, we consider only unigrams—for example, the terms “United States” and “Islamic State” are tokenized and lemmatized to “united”, “islamic”, and “state”. Although this is not ideal, we observe that this does not have a drastic impact on topic interpretability or separation.

The second potential limitation pertains to model evaluation. Although there exist clean, well-tested implementations of popular topic model quality metrics such as Pointwise Mutual Index (PMI) and UMass’ topic coherence (Mimno et al., 2011), Spark’s LDA implementation does not come with these metrics built in. In addition, Spark’s distributed nature, as well as its interface with the Java Virtual Machine mean that it is non-trivially complicated to implement custom user-defined metrics in Python. Due to these complexities, we evaluate topic quality using Spark’s existing “perplexity-bound” measure, as well as our own subjective evaluations of topic word intrusion and human interpretability.

Finally, with respect to reproducibility, a common practice to reproduce results in LDA is to fix the random seed across runs. This should in principle work well, since it ensures that the Dirichlet samples are drawn from the same random distribution across runs. However, because Spark is a distributed system, there is an additional layer of randomness in the methodology, i.e., that of the distributed system itself. Due to the way Spark distributes data, the order of samples passed to an executor cannot be guaranteed, nor can the same random number generator seed be guaranteed on each executor. As a result, this stage of the process is non-deterministic. This causes a very minor deviation in the topic keywords discovered across multiple runs, even with the same random seed on the exact same data. However, our numerous experiments over multiple months of news data confirm that this does not affect topic interpretability or the overall robustness of the process.

### Future Directions

A study of bias is not complete unless the full gender spectrum is considered, as well as other forms of diversity such as status as visible minority, disability, or age. Our language-based analyses rely on names to assign gender to individuals and are not well suited to study such forms of diversity. Additionally, potential harms from assigning specific labels to individuals need to be considered (Scheuerman et al., 2019; Blodgett et al., 2020). Nevertheless, studies of diversity in the news are valuable. Having a clear picture of the current levels of representation is the first step towards addressing any imbalances.

One area we are mindful about is the relative lack of work that studies topic modelling and gender disparities in languages other than English. The Gender Gap Tracker project has also been collecting news articles from Canadian French-language news outlets since October 2018, which we have not yet analyzed. Research on morphologically rich languages such as Russian or Swedish has shown that effective pre-processing techniques, such as lemmatization, can greatly improve topic interpretability (May et al., 2016; Devinney et al., 2020). Studying the relative benefits of stemming/lemmatization prior to topic modelling for French news articles would yield not only interesting methodological insights, but also reveal whether the same gender disparities we observed in English articles are present in French.

Due to the size and richness of data in the Gender Gap Tracker, we believe that a deeper analysis of gendered language by topic can yield further interesting insights (Hoyle et al., 2019). For instance, we are interested in studying whether quotes by men and women are presented differently in terms of endorsement or distance (*stated* vs. *claimed*) or in the way that the speaker is introduced, as an expert or merely a source. Analyses of transitivity structure in clauses can yield insights about the type of roles women are portrayed in (Power et al., 2019) and would complement the dependency analyses presented here. In general, the methodology for capturing male and female corpora per month can be deployed for any analyses of gendered language. While we plan to pursue such additional areas of inquiry, we also invite researchers to join in this effort.

## Conclusion

We analyzed a large news corpus to understand the relationship between topics in the news and the gender of those quoted. We also perform corpus-based language analyses using each article’s dominant topic distribution, confirming our hypothesis that articles that quote more men than women, on average, tend to use different words and verb-object combinations.

Our results, which consistently show that women are quoted more frequently in topics related to lifestyle, healthcare, and crimes and sexual assault, are, unfortunately, not unexpected. Multiple studies have found that women’s voices tend to be relegated to the domestic sphere and traditional female-dominated areas (Ross and Carter, 2011; Goodall, 2012; Shor et al., 2015; Ross et al., 2018). Specifically within the news domain and focusing on women as experts, women’s expertise is more likely to be found in “the sphere of the private, emotional and subjective” (Kassova, 2020b). Kassova also found that women are more central in crime and celebrity stories and less prominent in political and financial news (Kassova, 2020b). Women tend to speak as citizens rather than experts and, when they speak as experts, they do so more often about health, a caregiving profession, than about politics (Ross et al., 2018). When women are represented in political news, the lens is often gendered (Vavrus, 2002). This is a form of framing effect (Lecheler and de Vreese, 2018), which has an influence on how the public views gender in general.

Balanced gender representation in the media is within our reach, if enough effort is devoted to this goal and if we incorporate accountability into the effort. The Gender Gap Tracker, together with the topic-based analyses presented here, paint a clear picture of inequality in gender representation in the media. Our hope with the Gender Gap Tracker’s dashboard is that it be used as an accountability tool to encourage and facilitate gender parity in sources. We have seen from other initiatives, such as the BBC’s 50:50 campaign, that accountability leads to better balance in sources (British Broadcasting Corporation, 2020). In our view, providing organizations with a visual means to narrow down which topics exhibit the strongest disparities can have a tangible impact on improving the gender balance of sources.

Our public dashboard (https://gendergaptracker.research.sfu.ca) is updated every month, providing numbers and visualizations that can help Canadian newsrooms and policy makers monitor whether progress is being made. We open-source our code and visualization framework, so that others can also apply this methodology.

## Data Availability

The data was downloaded from public and subscription websites of newspapers, under the “fair dealing” provision in Canada’s Copyright Act. This means that the data can be made available only for private study and/or research purposes, and not for commercial purposes. As such, the data will be made available in a repository hosted by Simon Fraser University, accessible after signing a license agreement. The code is available on GitHub under a GNU General Public License (v3.0). The authors of this paper are co-contributors to the code base, hosted in the following repository: https://github.com/sfu-discourse-lab/GenderGapTracker.

## Footnotes

1
https://gendergaptracker.informedopinions.org/

2 We calculated the lengths on a random sample of 250 articles per outlet.

3
https://spark.apache.org/

4
https://databricks-prod-cloudfront.cloud.databricks.com/public/4027ec902e239c93eaaa8714f173bcfc/3741049972324885/3783546674231782/4413065072037724/latest.html

5
https://www.sfu.ca/research/supercomputer-cedar

6
https://databricks.com/blog/2015/09/22/large-scale-topic-modeling-improvements-to-lda-on-apache-spark.html

7
https://gendergaptracker.research.sfu.ca/apps/topicmodel

8
https://spacy.io/

9
https://github.com/explosion/spacy-lookups-data
