# Supplementary material for: Gender Bias in the News: A Scalable Topic Modelling and Visualization Framework
Source: Front Artif Intell. 2021 Jun 16;4:664737. doi: 10.3389/frai.2021.664737 (PMC8242240; doi:10.3389/frai.2021.664737)
Supplement: Supplementary file 1 [file Presentation1.pdf]

Supplementary Material for
Gender bias in the news:
A scalable topic modelling and visualization framework

Prashanth Rao,[1] Maite Taboada[1*]

[1]Discourse Processing Lab, Department of Linguistics, Simon Fraser University, Burnaby, BC, Canada

***Correspondence:** Maite Taboada, mtaboada@sfu.ca

Frontiers in Artificial Intelligence May 2021, Volume 4, Article 664737
https://doi.org/10.3389/frai.2021.664737

1 Background on topic modelling

In this section, we highlight some key aspects of topic modelling via Latent Dirichlet Allocation (LDA) that are relevant to our study. For a more detailed survey of various parametric and non-parametric probabilistic topic models, see Blei (2012).

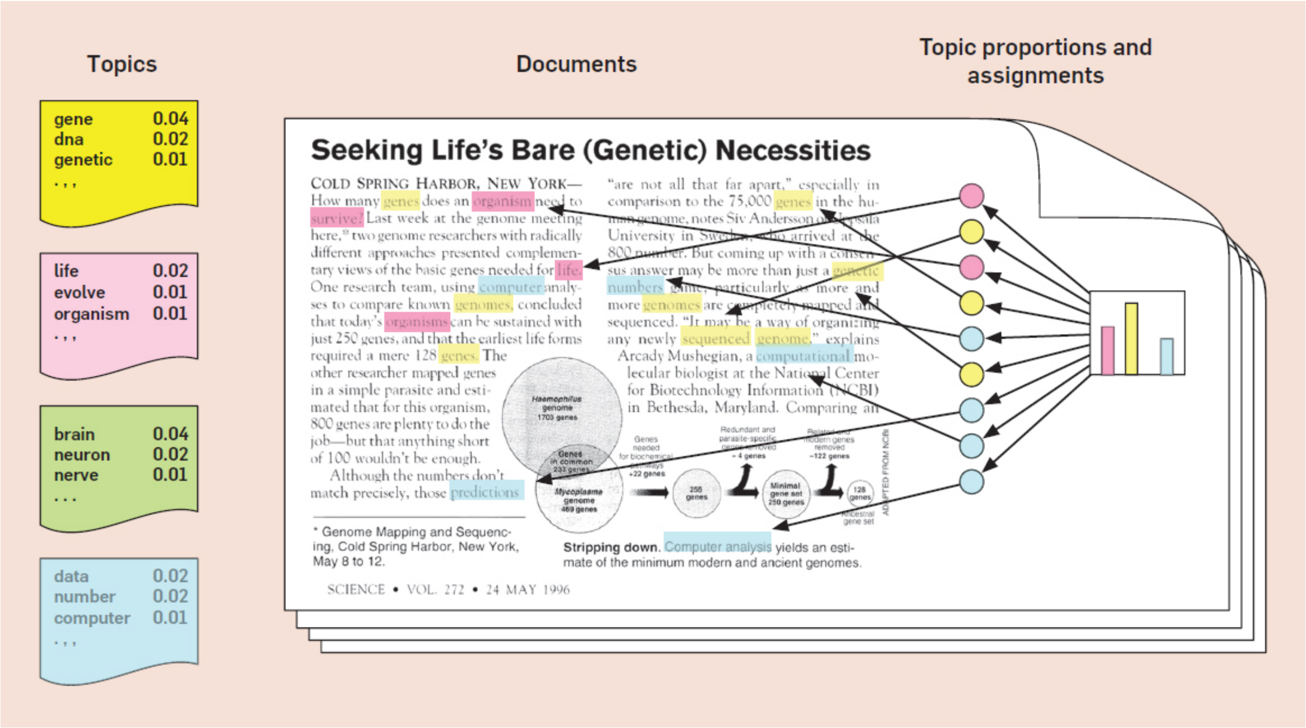

FIGURE S1 | Intuition behind LDA (Blei, 2012).

Figure S1 shows the intuition behind how an LDA model is applied in the real world. A fixed number of topics exist for the whole corpus, with each topic viewed as a distribution over words. Each document is viewed as a distribution over a fixed number of topics, which are themselves composed of words from a particular topic.

The following are key qualities of LDA that are relevant to the topic modelling methodology used in this study:

- It is a **generative probabilistic** model: The data in a corpus, i.e., the observed variables, are treated as though they arise from an imaginary random process that includes hidden (latent) variables.
- It is a **Bayesian** model: The generative process defines a joint probability distribution over both the observed and hidden random variables. This joint distribution is used during data analysis to compute the *posterior* distribution of hidden variables given the observed variables.
- It is a **mixed-membership** model: Each document exhibits multiple topics in different proportions, and each topic can exhibit words that also occur in other topics.
- It is a **bag-of-words** model: LDA does not consider word order in its distributions. This is quite sufficient for a coarse-grained semantic understanding of topic content over a large corpus.

1.1 Latent variables

The joint probability distribution of a topic mixture θ, a set of N topics z and a set of N words w is formulated as follows (Blei et al., 2003).

$$p(\theta, z, w \mid \alpha, \beta) = p(\theta \mid \alpha) \prod_{n=1}^{N} p(z_n \mid \theta)\, p(w_n \mid z_n, \beta) \tag{1}$$

$$\theta \sim Dirichlet\ (\alpha) \tag{2}$$

A major assumption in LDA is that the dimensionality k of the Dirichlet distribution (and thus the number of topics) is known and fixed beforehand by the user. In practice, determining the number of topics is a heuristic exercise (Zhao et al., 2015).

Because LDA is framed as a Bayesian problem, the key issue that needs to be resolved is one of inference, i.e., computing the posterior distribution of the hidden variables. Unfortunately, this distribution is intractable due to implicit coupling between θ and β in the summation over latent topics. The solution to this intractable problem was proposed by Blei et al. (2003)—an approximation called *variational inference* that closely matches the true posterior is used instead.

$$q(\theta, z \mid \gamma, \phi) = q(\theta \mid \phi) \prod_{n=1}^{N} q(z_n \mid \phi_n) \tag{3}$$

According to this formulation, new "free" variational parameters are introduced (the Dirichlet parameter γ and the multinomial parameter ϕ). This reframes the inference problem as an optimization problem that seeks to minimize the KL-divergence[1] between the variational distribution and the true posterior (Blei, 2012).

[1] In information theory, KL-divergence is the measure of the distance between two probability distributions.

Due to further mathematical complexities imposed by the mixture model setting and the presence of sparsity (a new document is very likely to contain words that did not appear in any documents in the training corpus), a Dirichlet *smoothing* step is applied. This introduces yet another parameter, η, which is used to smooth the free variational parameters. This ultimately influences the words distributed over topics through the β parameter.

$$\phi \sim Dirichlet\ (\eta) \tag{4}$$

In summary, an LDA algorithm learns the below hidden (latent) variables:

- α: Parameter that governs the topic distribution for each document.
- η: Parameter that governs the word distribution for each topic.
- θ: Random matrix $\theta_{i,j}$ representing the probability of the ith document containing the jth topic.
- β: Random matrix $\beta_{i,j}$ representing the probability of the ith topic containing the jth word.

Note that the α and η parameters are not necessarily scalars or symmetric vectors—in practice, they are modelled as asymmetric vectors to improve the stability and fitting accuracy of the algorithm.

1.2 Sampling vs. variational inference

Some implementations of LDA use a sampling-based approach to compute the approximation of the true posterior. The most common sampling method used is *Gibbs sampling*, in which a Markov chain of random variables is constructed with each variable dependent on the previous ones—the limiting value of this distribution equals the true posterior. The algorithm is run on the Markov chain defined on the hidden variables for a particular corpus and a number of samples are drawn using a *Markov Chain Monte Carlo* algorithm, following which the approximate distribution is constructed from the collected samples. While sampling-based methods are guaranteed to be identical to the true posterior under limiting conditions and can produce less biased results overall, they are quite computationally expensive and do not scale as well as variational Bayes methods do, as the corpus grows in size.

Due to the volume of data in the Gender Gap Tracker, we chose to avoid working with Gibbs sampling altogether. Instead, we use a *variational Bayes* inference model as implemented in Apache Spark[2] for all our experiments.

1.3 Expectation maximization vs. online variational Bayes

The variational method proposed by Blei et al. (2003) transforms a Bayesian inference problem to an optimization problem, i.e., an *expectation maximization* (EM) procedure that maximizes a lower bound with respect to the model parameters. However, due to the complex latent spaces typically seen in real-world corpora, it is quite common for variational EM methods to get stuck in local optima, resulting in the algorithm converging too slowly toward a global optimum or not at all.

An improved algorithm called *online variational Bayes* was developed to address this problem (Hoffman et al., 2010). The creators of this method note that, although variational Bayes (VB) inference methods are significantly faster than Gibbs sampling, they also suffer from computational difficulties for very large datasets. This is primarily because a standard VB algorithm must regularly switch between analyzing each observed batch and updating the dataset-wide variational parameters,

[2] Large scale topic modeling: Improvements to LDA on Apache Spark

which does not scale very well with enormous datasets consisting of millions of documents. The 'online' VB algorithm achieves faster convergence to a global optimum through stochastic optimization. Moreover, online VB does not locally store the documents—each document arrives in a stream and can be discarded after use, greatly improving its speed and performance.

Due to the massive efficiency gains seen with online VB, we opt to use this method as implemented in Apache Spark for all our topic modelling experiments.

2 Additional experiments

Below, we explain the results of some additional experiments we ran in order to arrive at our final methodology described in the paper.

2.1 Number of topics

Keeping all other hyperparameters constant, we train three topic models varying just the topic number ($k = 10, k = 15$ and $k = 25$) on **one month's worth** of news articles from seven mainstream Canadian English-language outlets. The month chosen was July 2019, consisting of approximately 29,000 articles of varying lengths. The resulting keywords are tabulated and human-labelled (using the top 15 words for the topic) to generate topic labels for the word distributions in each case. A summary of this comparison is shown in Table S1.

TABLE S1 | Comparison of topic labels for 10, 15 and 25 topics. Red topics are those that are repeated (with similar keyword distributions). Blue topics are those that are nearly the same across all three cases.

25 topics	15 topics	10 topics
Accidents and fire incidents		
Arts and entertainment		
Aviation incidents		
Business and market events		
Cannabis and health	Accidents and aviation incidents	
Community and indigenous programs	Arts and entertainment	
Crime and police investigations	Business and market events	Accidents and aviation incidents
Crime and police investigations	Crime and police investigations	Arts and entertainment
Education programs	Federal election campaign	Business and market events
Education programs and research	Healthcare	Crime and police investigations
Federal election campaign	Indigenous policy/government	Federal politics and elections
Healthcare	International protests and violence	Healthcare
Highway safety	Legal issues and court cases	Legal issues and court cases
Legal issues and court cases	Provincial projects/planning	Provincial projects and planning
Local food, restaurants and shopping	Sports (mainstream)	Sports (mainstream)
Local politics	Sports (summer)	US politics
Maritime events and updates	US politics	
Provincial energy budgets/planning	Weather and parks	

Provincial energy budgets/planning	World politics	
Sports (mainstream)		
Summer festivals and concerts		
US politics		
Weather and parks		
World politics		
World politics		

We know from the real world that articles published by news outlets fall into broad categories, such as sports, business, or politics. Our goal with this experiment is to judge the level of granularity in our model's discovered topics, and whether this is sufficient for our purposes in studying the relationship between topics and the gender distribution of people quoted. As can be seen in Table S1, all three cases show a good degree of topic separation, meaning that the model is capturing realistic themes from real-world news categories. It is important to note, however, that a topic model does not capture semantics of any kind, so the topics themselves may not perfectly correspond with a news outlet's categories from the real world (e.g., international news, business, or sports).

The labels in Table S1 indicate that, when 25 topics are used, a certain amount of repetition is present. On the other hand, with 10 topics, some important topics that may emerge in a given month, but not be present across time, are lost. We have found those two trends in multiple experiments with the 25-15-10 topic numbers. Due to issues with repetitive or non-existent topic labels and the difficulty of labelling a large number of topic keywords by hand, all our experiments going forward use **15** topics ($k = 15$). Coincidentally, Devinney et al. (2020) also found that 15 topics was a suitable number for their topic analyses of news articles.

2.2 Random seed

Because of the distributed nature of Spark, it is difficult to ensure that the same random number generator gets used across all executors, or that the order of samples being fed to the executor is fixed during model training. To test this, we run some experiments to study the stability of the LDA model over multiple runs for the month of March 2019. This particular month was chosen because it exhibits some interesting events that were of international importance, such as the aftermath of the Boeing 737 Max aviation disaster,[3] as well as the New Zealand mosque shootings.[4] Our goal is to see whether multiple LDA models with different random seeds can consistently capture the thematic structure of such events.

We first vary the random seed in Spark to three different (arbitrarily chosen) values: 1, 99, and 340573. The resulting topics, as interpreted by a human, are shown in Table S2. Note that, although the resulting topic word distributions are not identical across the different models, some domain knowledge of key world events that month, combined with some subjective judgement are sufficient to label the topics. It is clear that certain key events covered in the news that month, including the New Zealand mosque shootings, the Boeing 737 Max aviation disaster, and the SNC-Lavalin political scandal, are well-captured in all three models. The non-deterministic nature of the LDA

[3] CBC News: Canada grounds Boeing 737 Max 8
[4] CTV News: PM Trudeau condemns fatal shootings at mosques in New Zealand

model in Spark does, however, tend to fuse together two different topics (or introduce new topics altogether) into the top 15 topics for a given month (e.g., 'Education & medical research').

Because we limit each model's results to just 15 topics (for ease of labelling), we do not expect that our methodology captures *all* possible topics for a given month. Our primary requirement is that larger, more important themes that compose a given month's timeline be captured as far as possible, which we observe is true in these results. We tested two other months of data using the same three random seeds and observed similar trends with minimal loss of topic interpretability (with small amounts of word intrusion resulting in the merging of multiple smaller topics in certain cases).

TABLE S2 | Comparison of topic labels using three different random seed values (March 2019). Topics marked in orange show slightly different word intrusion and topic separation across cases.

Random seed: 1	Random seed: 99	Random seed: 340573
Arts & entertainment	Arts & entertainment	Arts & entertainment
**Boeing 737 Max aviation disaster**	**Boeing 737 Max aviation disaster**	**Boeing 737 Max aviation disaster**
Business & market events	Business & market events	Business & market events
Crime & police investigations	Crime & police investigations	Education programs & budgets
Education & medical research	European politics	Federal politics
Community infrastructure	Federal politics	Healthcare & medical research
Lifestyle	Healthcare & medical research	Legal & court cases
**New Zealand mosque shootings**	Lifestyle	Lifestyle
Provincial politics & programs	**New Zealand mosque shootings**	**New Zealand mosque shootings**
Severe weather updates	Provincial politics & programs	Provincial politics & programs
**SNC-Lavalin scandal**	Severe weather updates	Severe weather updates
Sports	**SNC-Lavalin scandal**	**SNC-Lavalin scandal**
Transport & highway safety	Sports	Sports
US politics	US politics	US politics
World politics	World politics	World politics

The next set of experiments are to study the repeatability of our modelling results. This time, we rerun the same model training step three separate times, using the same random seed of 1 (see Table S3).

TABLE S3 | Comparison of topic labels obtained over three runs of a single random seed (March 2019). Topics marked in orange show slightly different word intrusion and topic separation across cases.

Random seed: 1		
**Run 1**	**Run 2**	**Run 3**
Arts & entertainment	Arts & entertainment	**Boeing 737 Max aviation disaster**
**Boeing 737 Max aviation disaster**	**Boeing 737 Max aviation disaster**	Business & market events
Business & market events	Business & market events	Community infrastructure
Crime & police investigations	Consumer products & technology	Crime & police investigations
Education & medical research	European politics	Federal politics
Community infrastructure	Federal politics	Healthcare & medical research
Lifestyle	Healthcare & medical research	Legal & court cases
**New Zealand mosque shootings**	Legal & court cases	Lifestyle
Provincial politics & programs	Lifestyle & education	**New Zealand mosque shootings**
Severe weather updates	**New Zealand mosque shootings**	Provincial politics & programs
**SNC-Lavalin scandal**	Provincial politics & programs	Severe weather & transport safety
Sports	**SNC-Lavalin scandal**	**SNC-Lavalin scandal**
Transport & highway safety	Sports	Sports
US politics	Transport & highway safety	US politics
World politics	US politics	World politics & violence

As expected, fixing the random seed does not result in perfect reproducibility of the topic labels across multiple runs. While this is not ideal, a closer inspection of the labels indicates that the majority of topics are retained across all cases (including the key transient events for the month, marked in bold). Certain topics exhibit a small amount of overlap, combining keywords from multiple topics (e.g., 'Lifestyle & education' and 'Severe weather & transport safety'). However, this only seems to occur for 'minor' topics that do not feature in that many articles overall (minor topics are those that have weak topic weight intensities across all outlets).

Based on the random seed experiments, we confirm that in Spark there is an inherent difficulty in producing deterministic topic model results, even with the same random seed on the exact same data. However, considering that our overall goal is to study topic gender breakdown on select topics that feature strongly for any given month, we find that the trade-off between the reproducibility and scalability using our methodology is a reasonable one.

Because our overall goal is to study the relationship between topics covered in the media and the gender of people quoted, we also look at the effect of time span considered on the topics discovered. We would expect that running a topic model on several hundred thousand articles representing news coverage over one year's time would yield quite different topic labels from one that runs on just a month's worth of data. To study this further, we trained a series of models, over a 1-month, 3-month, 6-month and 12-month period.

Table S4 | Results for 12-month topics (April 2019–March 2020) and 6-month topics (October 2019–March 2020).

12 months	6 months
Arts and entertainment	Arts and entertainment
Business and market events	Business and market events
Community infrastructure	Consumer products, restaurants, and services
Consumer products, restaurants, and services	Crime and police investigations
Crime and police investigations	Federal politics and election campaign
Federal politics	Government policy
Government policy and human rights	Government policy and human rights
Healthcare and Covid-19	Healthcare and Covid-19
Provincial education policy and programs	Healthcare and medical research
Provincial projects and planning	Local businesses
Public affairs and unions	Provincial education policy and programs
Public events	Sports
Sports	US politics
US politics	Weather and natural disasters
World politics	World politics

Our experiment looks at the topic coverage before and during the COVID-19 pandemic that emerged in early 2020. The 12-month period considers all articles between April 2019 and March 2020, while the 6-month period considers all articles between October 2019 and March 2020. Table S4 shows the human-labelled topics from these two periods. Both periods largely reveal themes that are regularly covered in the news, such as 'Business and stock market', 'Arts and entertainment', 'Sports' and 'Federal politics'. It is interesting that the term '*Covid-19*' appears in the topic distributions even for the 12-month span dating back to April 2019—this is primarily because COVID-19 was a global crisis that dominated news coverage in Canada through the early period of 2020, making its terms co-occur very frequently with the 'Healthcare' word distribution. This is an undesirable result, as it makes it sound like COVID-19 was present going back to April 2019, which is not the case. Looking deeper at the remaining word distributions and their associated topic labels, we find that no fine-grained labels exist over these large time periods. Smaller and more transient events, expectedly, remain absent from the topic labels for this long a time span.

TABLE S5 | Results for 3-month (January–March 2020) 1-month topic model experiments (March 2020). Orange topics are more localized in time, representing more specific events or issues.

3 months	1 month
Arts and entertainment	Arts and entertainment
Business and market events	Business and market events
Community infrastructure	Covid-19 and healthcare guidelines
Coronavirus outbreak	Covid-19 and local communities
Covid-19 healthcare initiatives	Covid-19 and provincial updates
Covid-19 jobs and education support programs	Covid-19 and travel
Crime and police investigations	Covid-19 healthcare and support programs
Event cancellations and postponements	Covid-19 tracking and updates
Federal politics	Covid-19 business and market impact
Healthcare and medical research	Crime and police investigations
Indigenous rights and government policy	Event cancellations and postponements
Iran aviation disaster and political events	Government policy and support programs
Provincial education policy and programs	Sports
Severe weather and travel safety	US politics
Sports	World politics

We then compare the topic distributions from a 3-month period (January 2019–March 2020) and the 1-month period through March 2020, as shown in Table S5. Unlike the longer time spans, topics from these periods show much more fine-grained labels. As expected, COVID-19 and its related terms dominate the distribution, but even within this larger context, the model is able to disambiguate keywords from more fine-grained themes, such as 'COVID-19 and travel' and 'COVID-19 business and market impact'. Key world events such as the Iran aviation disaster in January 2020, including the political friction between the US and Iran in January/February 2020 emerged as a topic for the 3-month period.

From our time span topic experiments, we observed that news outlets typically spend a few days or weeks focusing on a particular event or issue, depending on its severity or importance. For transient events such as aviation or natural disasters that have a big impact on local communities, we believe that it is both insightful and important to model topics over shorter time spans for our source gender analysis. As a result, we settle on a **monthly** topic modelling pipeline for our further analyses and visualizations.

3 Topic labelling guidelines

In this section, we highlight some guidelines we used to generate human labels for each topic's keywords. Our topic model pipeline is designed as a monthly semi-automated process. On the first day of every month, a new topic model is trained on the previous month's English-language articles from seven outlets. The top 15 topic words for each topic (along with their topic weights) obtained from the LDA model are written to a database, following which they are human-labelled and visualized in greater detail. We maintain a fixed value of **15** topics a month for consistency across months and ease of labelling. The guidelines we use are detailed below, and experiments over the last

few months have shown that they produce labels that are reliably reproduced by multiple human annotators, over multiple rounds of topic modelling.

3.1 Naming patterns

We adopt a flexible topic naming pattern, in which a given distribution of keywords is interpreted (as best as one can) based on the larger themes that the words cover. Because not all topics can be described in 3–4 words, we occasionally use up to 5–6 words to label a topic more clearly. Some examples are shown in Table S6.

TABLE S6 | Typical naming patterns used in topic labelling.

Keywords	Topic label
vote, party, election, candidate, voter, campaign, liberal, poll, leader, political, quebec, seat, conservative, support, tory	Federal & provincial election campaigns
police, officer, floyd, protest, death, rcmp, charge, george, arrest, incident, black, protester, force, street, investigation	George Floyd protests & police investigations

3.2 Specificity

Rather than fixating too much on a single word (or pair of words) to identify a topic, we instead look at entire groups of words to identify larger themes. As an example, consider the keywords '*alberta, oil, price, energy, gas, industry, workers*'. It is clear from the keywords that there is a strong focus on energy as well as the oil and gas sector and its workers, so rather than choosing a vague label such as 'Provincial policy', we assign it the label 'Energy policy and jobs'. It is important to remember that there is no hard and fast rule to assigning good labels—this is ultimately down to subjectivity, domain knowledge, and human judgement.

In certain months, we observe keywords from different subtopics appearing across multiple topics—for example, different kinds of sports. We avoid assigning the exact same topic label, i.e., 'Sports' to such cases. To be as specific as possible, we disambiguate the names of the sports by inspecting the keywords and labelling them explicitly, for example, 'Sports (Grey Cup & CFL)' and 'Sports (Hockey & basketball)'.

3.3 Topic label reuse

Certain topics with similar keyword distributions appear again and again, regardless of the month of the year. Whenever possible, we reuse past topic labels for word distributions that are quite similar (for the most part), as shown in Table S7. This helps maintain consistency across months and allows for easier comparison of topic trends over time.

TABLE S7 | Repeating topic labels we tend to reuse (for similar keyword distributions).

Topic label	Typical keywords
Arts & entertainment	*book, film, black, part, award, gallery, art, music, movie, toronto*
Business & market events	*company, market, bank, trade, sell, buy, billion, stock, investor*
Community infrastructure	*province, cost, million, project, housing, budget, pay, transit, road*
Crime & police investigations	*police, officer, rcmp, investigation, victim, arrest, kill, die, suspect*
Lifestyle	*child, woman, mother, young, house, daughter, community, experience*
Federal politics	*party, trudeau, liberal, conservative, candidate, vote, ndp, campaign*
Healthcare & medical research	*health, study, research, care, patient, hospital, medical, drug, case*
Highway & transport safety	*driver, car, truck, system, safety, traffic, drive, vehicle, crash, injury*
Jobs, education & worker unions	*worker, community, union, school, student, project, strike, board*
Legal & court cases	*court, case, judge, lawyer, charge, decision, justice, legal, appeal*
Sports	*game, team, season, player, hit, point, shoot, goal, coach, win*
US politics	*president, unite, trump, state, house, republican, administration*
Severe weather updates	*snow, park, water, fire, winter, road, ice, high, heat, temperature*
World politics	*country, international, minister, national, border, china, unite, state*

4 Quantitative metrics

In this section, we describe the various quantitative metrics we use in our topic keyword and language analyses. Using the results from LDA as described in the main paper, we are able to characterize each individual news article in our corpus for a particular month as belonging to a distribution over topics. Mathematically, this means that the topic modelling process returns a one-dimensional vector of length 15 (we only model a maximum of 15 topics each month) for each document, where each component of the vector represents how strongly or weakly that topic's keywords are associated with that document. Some examples of how these results are represented in our database are shown in Table S8.

TABLE S8 | Example output snippet from topic modelling (Bold weights indicate dominant topics).

Article ID	Outlet	# Female sources	# Male sources	Topic weight distribution [t1, t2, … , t15]
1	CBC News	1	0	[**0.996**, 0.002, … , 0.0001]
2	Huffington Post	3	1	[0.0002, **0.992**, …, 0.0001]
3	The Globe and Mail	0	2	[0.0001, 0.0003, …, **0.675**]
4	CTV News	1	3	[0.0001, **0.995**, …, 0.0003]
...	...	...	...	...

We know from our analysis of statistics from the Gender Gap Tracker that, on average, there are roughly 3–4 times more articles with a majority of male sources than those with a majority of female sources ('majority' here means at least one more source from one gender than the other). As a result, rather than looking at raw counts of articles and which topics dominate in them, we compute the **mean** of the topic distributions over the outlets and/or dominant gender quoted. Because we have at least a few thousand articles each month (with a few hundred, at least, per news outlet) that quote more female sources than male, we believe we have a large enough sample size to draw reasonable conclusions from.

The topic modelling results, once computed, are visualized on an interactive dashboard (https://gendergaptracker.research.sfu.ca/apps/topicmodel) for easy exploration.

4.1 Topic intensity

As a first step, we are interested in answering the question: *Which topics were covered more extensively by each outlet in a particular month*? To do this, we first group our results by outlet, and compute the element-wise mean topic weight for all articles from that outlet. This returns a [15 x 7] matrix, representing the mean topic weights over 15 topics for articles from all seven English news outlets in that month. This matrix is easily visualized as a heat map as shown in Figure S2.

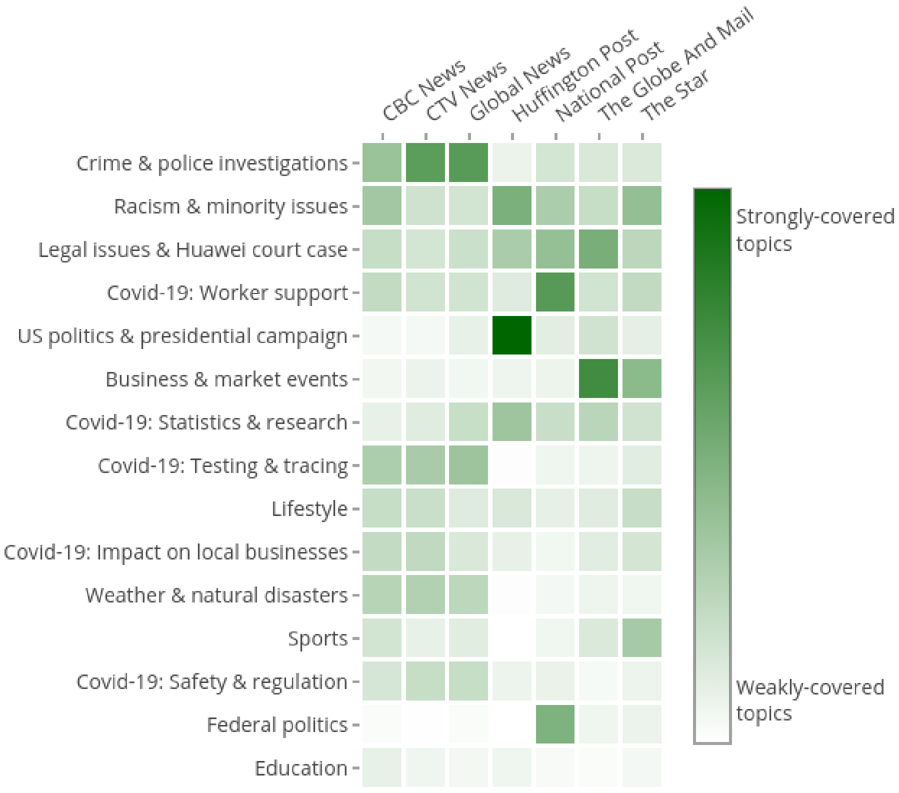

FIGURE S2 | Heat map of mean topic intensity per outlet for news articles in July 2020.

The heat map of mean topic intensity is ordered by the sum of means for all outlets, with the most strongly covered topic for that month (on average, across all outlets) appearing on top. From Figure S2, for the month of July 2020, it is clear that a large proportion of articles contain keywords

pertaining to crime, police investigations, and racism and minority issues. Relatively fewer articles contain keywords pertaining to the lifestyle, sports, or education topics.

4.2 Topic gender prominence

In order to study the difference in representation between male and female sources for each topic discovered, we perform one additional step prior to aggregation. The corpus of articles shown in Table 11 is separated into two smaller corpora—those with majority male sources, and those with majority female sources.[5] The majority condition is easily calculated by comparing the columns containing the source counts for either gender from Table 11. We refer to these corpora as the **female** and **male** corpora from this point on**.** Next, we once again group our results by outlet and aggregate the topic weights, but this time, we do so for *each corpus* (with male/female majority sources) separately. This results in two [15 x 7] matrices (one for either corpus), each representing the mean topic weights over 15 topics for articles from the seven outlets.

4.2.1 Per-outlet gender prominence

Here, we introduce the term 'gender prominence' to help disambiguate how different topics are related to the number of female/male sources quoted. For the purposes of this study, we define gender prominence as the difference in mean topic weights between the female and male corpora for a given topic. A topic is categorized as having male prominence if the mean topic weights from the male corpus are greater than those from the female corpus. Similarly, a topic can be said to exhibit female prominence if the mean topic weights from the female corpus are greater than those for the male corpus. Mathematically, this is calculated as the element-wise difference between the two [15 x 7] topic weight aggregation matrices. A positive difference indicates that the topic exhibits female prominence, whereas a negative difference indicates male prominence. Figure S3 showcases this result as a heat map for the topics discovered in July 2020.

[5] We define the 'majority' condition here as any case where the number of sources from one gender is **one or more** greater than the number of male sources from the other gender. For example, an article with 3 female sources and 2 male sources is categorized as 'female-majority' and is assigned to the female corpus.

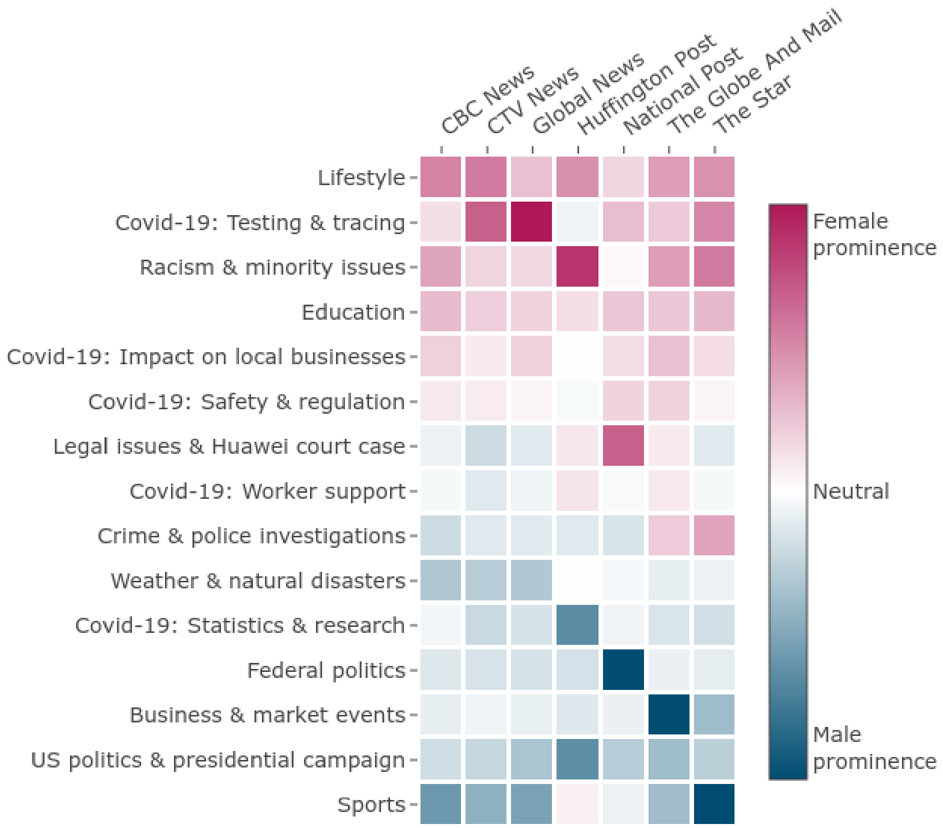

FIGURE S3 | Heat map of mean topic gender prominence per outlet for news articles in July 2020.

The heat map of topic gender prominence uses a divergent colour scale. Topics that are at the extreme ends of the heat map ('Lifestyle' at the top and 'Sports' at the bottom) exhibit the strongest disparity per-topic in the mean topic weights of the female/male corpora. For July 2020, the 'Lifestyle' topic was much stronger (i.e., had a higher mean topic weight) in the female corpus, leading to a greater positive difference (red) between the topic weight matrices. Conversely, the 'Sports' topic was much stronger in the male corpus, leading to a greater negative difference (blue) between the topic weight matrices.

Note that in the gender prominence heat map, a zero value (white) indicates neutrality. A topic can be 'gender-neutral' in one of two ways. First, there might exist true parity in topic intensity between the two corpora (with female/male-majority sources), where both corpora exhibit the same mean topic weight, leading to their difference being zero. Alternatively, the topic might just have been non-existent for that particular month, resulting in both the male and female corpora showing a zero topic weight for that topic.

4.2.2 Overall gender prominence

In addition to the heat maps, we also provide bar charts of mean topic weights across *all* outlets for the female and male corpora, as shown in Figure S4. The data for these plots is obtained by simply aggregating topic weights over all articles in either corpus (i.e., with female or male-majority sources).

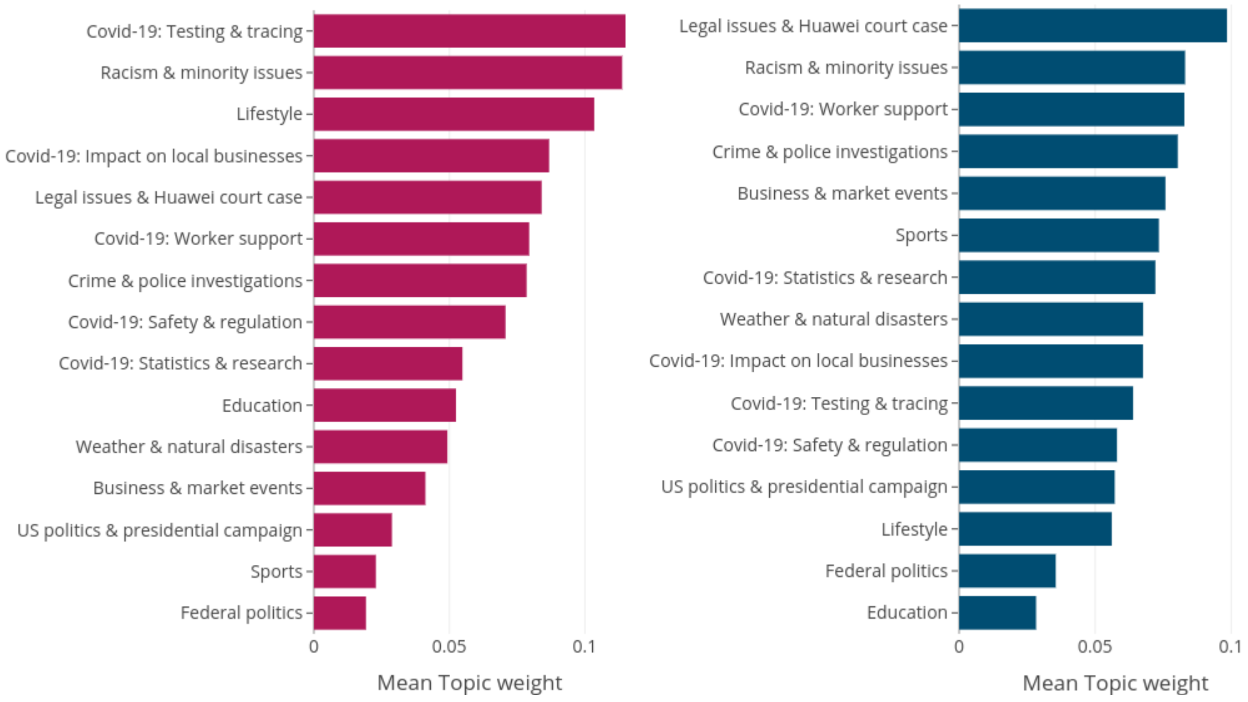

FIGURE S4 | Bar plots of topic gender prominence for news articles over all outlets in July 2020.

The overall gender prominence plots shown are ranked in decreasing order of mean topic weights. Higher values indicate that the topics were particularly strongly covered for the corpus in question. For July 2020, we can see in Figure S4 that topics such as ‘COVID-19: Testing & tracing’ and ‘Lifestyle’ show the highest gender prominence in the female corpus, whereas topics such as ‘Legal issues & Huawei court case’, ‘Business & market events’, and ‘Sports’ show higher gender prominence in the male corpus.

Interestingly, the variance between the top and middle topic weights is much greater for the female corpus than for the male corpus. We think this is primarily due to the fact that many, many more articles exist that quote more men than women,[6] so it is natural that men’s voices are more equitably distributed across the topics. We observe a similar trend for almost all the months for which we have data, meaning that there could exist a correlation between the gender distribution of sources in an article and the likely content it covers.

5 How we define male and female corpora

The heat maps shown in Section 4 provide a high-level overview of which topics tend to show male or female prominence over time. However, to gain an understanding of *why* certain topics in certain months show specific gender distributions in their top quoted sources, a deeper linguistic analysis is required. Because we already divide our news article content into two separate corpora based on which gender is most quoted, our scenario is well-suited to *corpus studies*, i.e., a set of techniques that are known to “help deconstruct hidden meanings and the asymmetrical ways people are represented in the press” (Caldas-Coulthard and Moon, 2010).

[6] In July 2020, there were 12,723 articles in the male corpus, and just 4,303 articles in the female corpus.

We first manually identify a topic of interest that exhibits strongly male or female gender prominence for a particular month that we want to explore in more detail. We then query that month's data from our database, sorted in descending order of topic weights for that topic (recall that we store the topic distribution vector for every article). Sorting the articles in this order puts all articles that are strongly related to that topic's keywords on top. The full corpus, in sorted order for a particular topic, is then split into two corpora, each with male-majority and female-majority sources. An illustration of this workflow is shown in Figure S5.

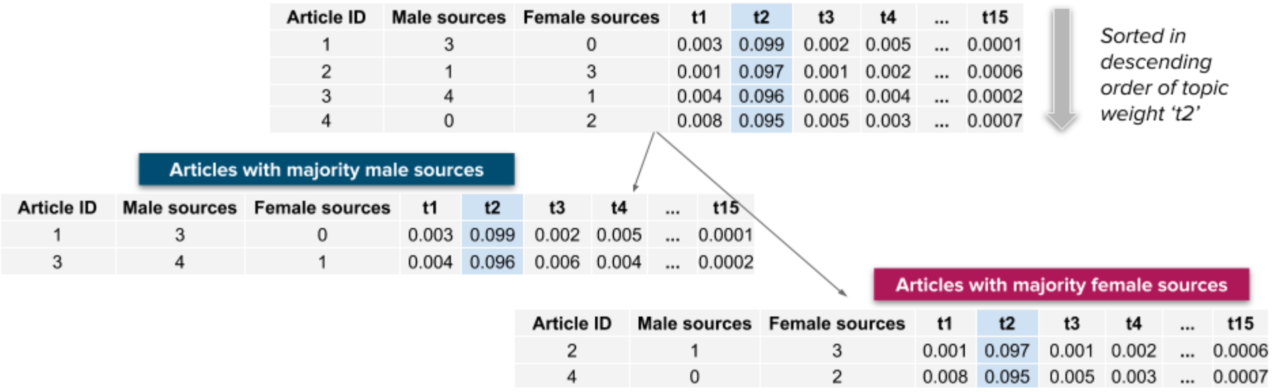

FIGURE S5 | Topic-wise sorting and article extraction for corpus analysis.

Once we have the two corpora, we then extract the full body article text (using the article IDs) for the top 200 articles in either corpus from our database. We chose 200 articles for empirical reasons—we observed that in most cases, the maximum topic weights for each article rapidly dropped to less than 0.5 after a few hundred samples (sorted in descending order of weights), so it didn't make sense to go too far down in the list of articles strongly associated with a particular topic.

Following these steps, we use the *'corpus-toolkit'* Python library (built on top of spaCy)[7] to perform keyness analysis and extract dependency bigrams, as described in the paper.

References

Blei, D. M. (2012). Probabilistic topic models. *Communications of the ACM*, 55(4), 77–84.

Blei, D. M., Ng, A. Y., & Jordan, M. I. (2003). Latent Dirichlet Allocation. *Journal of Machine Learning Research*, 3(Jan), 993–1022.

Caldas-Coulthard, C. R., and Moon, R. (2010). 'Curvy, hunky, kinky': Using corpora as tools for critical analysis. *Discourse & Society* 21 (2), 99–133.

Devinney, H., Björklund, J., & Björklund, H. (2020). Semi-Supervised topic modeling for gender bias discovery in English and Swedish. *Proceedings of the Second Workshop on Gender Bias in Natural Language Processing*, 79–92.

Hoffman, M., Bach, F. R., & Blei, D. M. (2010). Online learning for Latent Dirichlet Allocation. *Advances in Neural Information Processing Systems*, 856–864.

Zhao, W., Chen, J. J., Perkins, R., Liu, Z., Ge, W., Ding, Y., & Zou, W. (2015). A heuristic approach to determine an appropriate number of topics in topic modeling. *BMC Bioinformatics*, 16, S8.

[7] https://kristopherkyle.github.io/corpus_toolkit/
